# Pannexin1 Channel Proteins in the Zebrafish Retina Have Shared and Unique Properties 

**DOI:** 10.1371/journal.pone.0077722

**Published:** 2013-10-23

**Authors:** Sarah Kurtenbach, Nora Prochnow, Stefan Kurtenbach, Jan Klooster, Christiane Zoidl, Rolf Dermietzel, Maarten Kamermans, Georg Zoidl

**Affiliations:** 1 Neuroanatomy, Faculty of Medicine, Ruhr University Bochum, Bochum, Germany; 2 Research Unit Retinal Signal Processing, the Netherlands Institute for Neuroscience, Amsterdam, The Netherlands; 3 Neurogenetics, Academic Medical Center, Amsterdam, The Netherlands; 4 Psychology, Faculty of Health, York University, Toronto, Ontario, Canada; University of Michigan, United States of America

## Abstract

In mammals, a single pannexin1 gene (Panx1) is widely expressed in the CNS including the inner and outer retinae, forming large-pore voltage-gated membrane channels, which are involved in calcium and ATP signaling. Previously, we discovered that zebrafish lack Panx1 expression in the inner retina, with drPanx1a exclusively expressed in horizontal cells of the outer retina. Here, we characterize a second drPanx1 protein, drPanx1b, generated by whole-genome duplications during teleost evolution. Homology searches strongly support the presence of pannexin sequences in cartilaginous fish and provide evidence that pannexins evolved when urochordata and chordata evolution split. Further, we confirm Panx1 ohnologs being solely present in teleosts. A hallmark of differential expression of drPanx1a and drPanx1b in various zebrafish brain areas is the non-overlapping protein localization of drPanx1a in the outer and drPanx1b in the inner fish retina. A functional comparison of the evolutionary distant fish and mouse Panx1s revealed both, preserved and unique properties. Preserved functions are the capability to form channels opening at resting potential, which are sensitive to known gap junction and hemichannel blockers, intracellular calcium, extracellular ATP and pH changes. However, drPanx1b is unique due to its highly complex glycosylation pattern and distinct electrophysiological gating kinetics. The existence of two Panx1 proteins in zebrafish displaying distinct tissue distribution, protein modification and electrophysiological properties, suggests that both proteins fulfill different functions *in vivo*.

## Introduction

Pannexin1 (Panx1) shares topological similarities with gap junction forming connexins (Cx), and functions as a large-conductance channel (for review see [1]). Panx1 is ubiquitously expressed in the CNS including sensory systems like the eye [2–4]. Panx1 channels open by purinergic receptor activation [5,6], increased intracellular calcium levels [6] and depolarization of the plasma membrane [7,8]. In the murine visual system, Panx1 is expressed in the inner and outer retinae. Panx1 expression dynamically changes between young and adult mice with a transient peak around birth that decreases in the adults [2,4]. In previous studies, we identified an ortholog of mammalian Panx1 in zebrafish (zfPanx1/drPanx1a) and described its expression in the fish retina [9–11]. The initial biophysical characterization together with the prominent localization of drPanx1a at the tips of horizontal cell dendrites led us hypothesize that drPanx1a might participate in ephaptic feedback mechanisms to process visual information in the outer retina [10,12]. A recent study provided evidence that this is may also be true for mouse Panx1 (mPanx1) [13]. However, the lack of Panx1 expression in the inner zebrafish retina is inconsistent with the spatial distribution of Panx1 found in mouse and rat retinae. Now, we report the cloning and functional characterization of a second zebrafish Panx1 protein, denominated drPanx1b. drPanx1b most likely originates from the third major teleost whole genome duplication (WGD) event occurring around 320 and 350 million years ago [14]. Initial analysis of the distribution of the two drPanx1s revealed that the *drpanx1a* gene seems to be expressed ubiquitously, similar to the mammalian Panx1, whereas *drpanx1b* shows a highly specific expression pattern in the brain. Both genes are expressed in equal amounts in the retina. 

Here, we demonstrate that drPanx1a and drPanx1b expression levels vary in different brain areas and that drPanx1b, like murine Panx1, is expressed in the inner nuclear and ganglion cell layer of the retina. Functional analyses comparing both drPanx1 proteins to mPanx1 revealed conserved and unique properties. The investigated Panx1 proteins form channels, which open under physiological conditions and are sensitive to know gap junction and hemichannel blockers, elevated intracellular calcium levels and ATP. Further, we demonstrate pH dependent Panx1 channel modulation, which is a novel form of Panx1 modulation. drPanx1b differs from drPanx1a and mPanx1 due to their more complex glycosylation putatively involving three N-glycosylation sites and by different electrophysiological gating kinetics. These results are of considerable importance in light of the anticipated roles of Panx1 in processing of visual information, complementing the hypothesized role of drPanx1a in feedback modulation the outer retina [10,12] with additional and potentially unique functions of drPanx1b in the inner retina. In summary, the coordinated expression of two Panx1 proteins suggests that both proteins could operate in distinct functional circuits adding functionality to physiological processes shaping visual output. 

## Materials and Methods

### Animals

Zebrafish (*Danio rerio*) of either sex were kept at 28°C in aerated tanks filled with tap water circulating through a bacterial filter system on a 12 h light-dark cycle. Animal handling and sacrificing were carried out according to the guidelines of the German Animal Protection Law in its present version and under the responsibility of the ethical committee of the Royal Netherlands Academy of Arts and Sciences acting in accordance with The European Communities Council Directive of 24 November 1986 (86/609/ EEC). Fish were sacrificed by trained staff after anesthesia with ethyl 3-aminobenzoate methane sulfonate (tricaine/MS-222 (Finquel); 0.01% w/v) and cervical dislocation. Isolation of animal tissues was kept to a minimum after mandatory notification of the Animal Welfare Officer and the Veterinary Office of the City of Bochum. 

### Plasmid constructs and site-directed mutagenesis

Full-length drPanx1a (amino acids (aa) 1-417; GI: 28856207) and full-length mPanx1 (aa 1-426; GI: 18043025) were cloned into the pEYFP-N1 expression vector (Clontech Laboratories Inc., Mountain View, CA, USA) as described [2,10,11]. Full-length drPanx1b (aa 1-422) was obtained by PCR amplification using adult zebrafish brain and retina cDNA as template. Oligonucleotides used: S1 5’-ATGGCTATAGCGCGGGTAGC-3’ and AS1 5’-CAGCGAAAGTCGTCTAAAGGCA-3’. The drPanx1b protein coding region was cloned into the pEYFP-N1 and pIRES2-mRFP expression vectors (generated by replacing EGFP by mRFP1 in pIRES2-EGFP (Clontech Laboratories Inc., Mountain View, CA, USA)) and the sequence compared to a reference sequence found in the NCBI database (NM_001100030.1, GI: 153791522). Expression of pEYFP-Panx1 constructs results in C-terminal EYFP-tagged Panx1 fusion proteins, while expression of pIRES2-mRFP1-drPanx1b leads to co-expression of untagged drPanx1b and mRFP1. Mutant pEYFP-drPanx1a and pEYFP-drPanx1b plasmids were generated using Transformer^TM^ Site-Directed Mutagenesis Kit (Clontech, Takara) according to the manufacturer’s protocol. Mutagenesis oligonucleotides used (mutated aa are depicted in bold and underlined):

N71K 5’-GTTTTCCTCCAACTAA**G**TTCACGATGAGACAG-3’,
N95K 5’-ATCACCCTTCAGAGAA**G**GAGACCTACAGCGCC-3’,
N246K 5’-GAGGTATATTAGTGAA**G**CAGAGTGAAGTGCC-3’,
drPanx1a-C76R 5’-GCGTTCTCATGG**C**GTCAGGCAGCTTACG-3’;
drPanx1a-C76K 5’-GCGTTCTCATGG**AAG**CAGGCAGCTTACG-3’;
drPanx1a-C76A 5’-CAGCGTTCTCATGG**GC**TCAGGCAGCTTACGTG-3’;
drPanx1a-C76E 5’-GCGTTCTCATGG**GAA**CAGGCAGCTTACG-3’;
drPanx1b-R75C 5’-AATTTCACGATG**T**G**C**CAGGCTGCGTATG-3’;
drPanx1b-R75K 5’-CTAATTTCACGATGA**A**ACAGGCTGCGTATGCG-3’;
drPanx1b-R75A 5’-CTAATTTCACGATG**GC**ACAGGCTGCGTATGCG-3’;
drPanx1b-R75E 5’-CTAATTTCACGATG**GA**ACAGGCTGCGTATGCG-3’

### Quantitative Real Time-PCR

Total RNA was extracted from adult fish, reverse transcribed and used in quantitative Real Time-PCR as described [9]. The primer pairs were: drPanx1a-S 5’-ACCTTCTTTGCCGCACCATCACTGT-3’, drPanx1a-AS 5’-AGGGCTGGAGGCACGGAGGAGTC-3’, drPanx1b-S 5’-TTCCTGGAGGAAAACCTGAGTGAGC-3’, drPanx1b-AS 5’-CCAAGAGTCCTGAGCAAACACATGG-3’, 18s-S 5‘-GAGGTGAAATTCTTGGACCGG-3‘ and 18s-AS 5‘-CGAACCTCCGACTTTCGTTCT-3‘ designed for drPanx1a, drPanx1b and 18s cDNA amplification. The conditions for qPCR and data analysis strategy have been reported [9]. All experiments represented three independent sets of samples analyzed in triplicates. Statistical analysis was performed using the Relative Expression Software Tool (REST) software and the student’s *t*-test.

### Cell culture, transient transfection and western blot

Neuroblastoma 2a (N2a) cells [15] were maintained and used for localization studies, dye uptake and electrophysiology as described [10,11]. Cells were transfected with 200-600 ng DNA and analyzed 48 h post transfection. Whole cell protein lysates (20 µg) from transfected N2a cells were separated by 10% SDS-PAGE, transferred to 0.2 µm Hybond-ECL nitrocellulose membrane (Amersham Biosciences, GE Healthcare, Freiburg, Germany) and processed as described [3]. Primary antibodies were diluted 1:1,000 (mouse anti-GFP, Roche; rabbit anti-GFP (FL), Santa Cruz) and 1:20,000 (mouse anti-β-actin; Sigma-Aldrich Chemie GmbH, Munich, Germany). The secondary antibodies (LI-COR Biosciences, St. Lincoln, NE, USA) were diluted 1:20,000 (goat anti-mouse IRDye680RD; donkey anti-rabbit IRDye680LT) or 1:15,000 (goat anti-mouse IRDye800CW). Signals were detected using the Odyssey® Infrared Imaging System (LI-COR Biosciences).

### Antibody preparation

The polyclonal rabbit anti-drPanx1a antibody has been described previously [10]. The antibody is specific for drPanx1a and does not cross react with drPanx1b. A chicken anti-drPanx1b antibody was produced and purified by the Davids Biotechnologie GmbH (Regensburg, Germany) against the aa sequence GHVLDLQPATRYDDLS (aa 309-325) representing a C-terminal sequence unique for drPanx1b. Despite extensive testing, the affinity purified IgY antibody failed in all western blot conditions tested. Specificity in immunohistochemistry was confirmed using brain and retina tissues known to express drPanx1b mRNA. Peptide blocking and control reactions without primary antibody complemented this attempt. 

### Immunohistochemistry and confocal microscopy

Eyes and brain were isolated from cervically transected adult zebrafish and processed for immunohistochemistry as described [16]. In short; eyes were fixed in 4% paraformaldehyde (pH 6.5) for 10-15 min and afterwards fixed in 4% paraformaldehyde (pH 10.4) for additional 10-15 min. Subsequently, eyes were rinsed in phosphate buffer (0.1 M, pH 7.4), cryoprotected in sucrose 12.5 % and 25 % in phosphate buffer and frozen in Tissue Tex. Finally, 10 µm sections were made. Sections were incubated over night with the chicken anti-drPanx1b antibody. The primary antibody was visualized by an anti-chicken antibody conjugated with Alexa488 (1:500; Jackson ImmunoResearch, Suffolk, UK), which was incubated at 36°C for 35 min. Sections were embedded with VECTASHIELD containing propidium oxide. Images were captured with an LSM 510 META system (Zeiss). For diaminobenzidine (DAB) staining using the VECTASTAIN Elite ABC Kit (Vector Laboratories, Burlingame, CA, USA), tissues were embedded into tissue wax (MEDITE, Burgdorf, Germany) and cut into 11 μm thick slices. Slices were deparaffinized and treated accordingly to the manufacturer’s guidelines. As primary antibodies, the rabbit anti-drPanx1a or chicken anti-drPanx1b (1 µg/ml) were used. The biotinylated goat anti-rabbit or anti-chicken (1:1000, AXXORA, Lörrach, Germany) were used as secondary antibodies. Pictures were taken with an Olympus BH-2 light microscope (Olympus Deutschland, Hamburg, Germany) and an Olympus DP71 camera using the *cellA* software. 

Confocal image analysis was performed on transiently transfected N2a cells grown on glass bottom culture dishes 48 h after transfection. During live cell recording cells were maintained in a standard physiological extracellular solution (SPES) composed of (in mM): 147 NaCl, 10 HEPES, 13 glucose, 2 CaCl_2_, 1 MgCl_2_, and 2 KCl, pH 7.4 and imaged using the LSM 510 META system (Carl Zeiss MicroImaging GmbH, Cologne, Germany) as described [16]. Image processing was performed with the LSM 510 META software.

### Dye uptake assay

Ethidium bromide (EtBr, AppliChem GmbH, Darmstadt, Germany) dye uptake assays using SPES (see immunohistochemistry) as recording solution were performed as previously described [10,11]. In three independent experiments, a total of n = 135 cells was analyzed for each condition. Inhibitory compounds (all from Sigma-Aldrich) were applied together with EtBr in their final concentration. SPES with different pH values was applied to test pH sensitivity by manipulating the extracellular pH. Digitonin application was utilized to induce maximal EtBr uptake at the end of each recording. This step proves that decreased fluorescent intensity in low extracellular pH experiments is not caused by hydrolysis of the EtBr molecule by acidic pH values. Fluorescence values after 5 min after EtBr application were subjected to statistical analysis. The fluorescence values of drPanx1a or drPanx1b expressing cells under control conditions were set to 100%. The averaged results are expressed as the means + standard error of the means (SEM). All statistical analyses were performed with Prism 5 (GraphPad Software, La Jolla, CA, USA) with a confidence limit for significance set at 0.05. The non-parametric Kruskal-Wallis test followed by a Dunn’s Multiple Comparison post-test was used for the analyses of three or more groups. For comparisons between two populations of data sets, the non-parametric Mann-Whitney rank sum test was performed. Gaussian distribution was not assumed. 

### Electrophysiology

#### Whole-cell patch clamp recordings

Whole-cell patch clamp recordings in the voltage clamp mode of transfected N2a cells were performed as described [10], using the identical set-up with SPES (see immunohistochemistry) as extracellular solution. The pipette solution contained (in mM): 130 K-gluconate, 2 Na-gluconate, 20 HEPES, 4 MgCl_2_, 4 Na_2_-ATP, 0.4 Na_2_-GTP, 5 mM EGTA, pH 7.2. The cells were voltage clamped to -30 mV before running the protocol. To characterize the Panx1 channel activity a preconditioning paradigm was established, allowing a standardized comparison of Panx1 channels before and after a voltage driven channel activation (modified from Gründken et al., 2011). The extracellular standard artificial cerebrospinal fluid (ACSF) contained (in mM): 124 NaCl, 2.69 KCl, 1.25 KH_2_PO_4_, 26 NaHCO_3_, 2 MgSO_4_, 2 CaCl_2_ and 10 glucose. The pipette solution contained (in mM): 130 potassium gluconate, 2 sodium gluconate, 20 HEPES, 4 MgCl_2_, 4 Na_2_ATP, 0.4 NaGTP and 0.5 EGTA, pH 7.3. Holding potential steps with a duration of 250 ms starting at -60 mV to +100 mV in 10 mV increments were applied (**I**), followed by three preconditioning 10 s depolarizing voltage ramps ranging from -60 mV to +80 mV with an inter-ramp-interval of 30 s at -60 mV. Directly after the third preconditioning ramp, the initially applied holding voltage steps (**I**) were repeated (**II**). EYFP expressing N2a cells served as a control.

#### Excised patch recordings

External and internal solution conditions for excised outside-out patch clamp recordings were adopted according to protocol B, Gründken et al. (2011). The glass coverslips were superfused and continuously gassed with carbogen at RT (5% CO_2_/95% O_2_). Pipettes were pulled to a average tip diameter of 1 µm with an input resistance of 10-15 MΩ [10,11]. Recordings were performed using a 10 GΩ head stage, with signals amplified and filtered at 0.15 kHz by a Warner PC-505B amplifier (Warner Instruments; Hamden CT, USA). 

#### Analyses of electrophysiological data

The current-voltage relation (I/V relation) was calculated from the membrane current response evoked by the depolarizing voltage step in protocol **I** and **II** of the preconditioning paradigm, by averaging the membrane current during the last 10 ms at the end of each voltage step. Liquid junction potential and holding currents were not corrected. The data were analyzed with WinWCP and Microsoft Excel. The average results are shown as the mean + the standard error of the mean (SEM). The statistical analyses using Prism 5 was performed as described for the dye uptake assays. Statistical analysis of single channel data was carried out with Sigma plot 11.0 software (Systat; Erkrath, Germany). The non-parametric Mann-Whitney rank sum test was used two compare two data sets. Electrophysiological results are presented as the mean + standard error of the mean (SEM) of the specific number of cells/membrane fragments. Single channel data were analyzed with WinEDRv3.2.6 data acquisition software (Strathclyde; Biologic, Knoxville TN, USA). 

### Phylogenetic analysis

Phylogenetic relationships were calculated using protein sequences to avoid errors due to multiple substitutions and homoplastic characters present in nucleotide sequences. Protein sequences were obtained from the NCBI database, and by performing various BLAST searches with multiple Pannexin and Pannexin consensus sequences using the DOE Joint Genome Institute (JGI) Portal [17], the *Branchiostoma floridae* v2.0 and the *Ciona intestinalis* v.2.0 assembly. Shark sequences were obtained by performing BLAST searches on http://esharkgenome.imcb.a-star.edu.sg. *Hydra magnipapillata* sequences were obtained from [18], and BLAST searches were performed on http://metazome.net/. Sequences shorter than 40 aa were excluded. Sequences were aligned using MAFFT [19] version 6.932 and the L-INS-I strategy. ProtTest [20,21] version 3.2 determined the JTT +G as the most likely model of protein evolution for the dataset, using the Baysian Information Criterion (BIC) and Decision Theory (DT) model (Akaike information criterion (AIC) suggested the JTT +G +F model, which did not change the tree topology substantially; data not shown). Phylogenetic tree calculations were performed with RAxML-HPC2 on XSEDE, on the CIPRES Science Gateway [22,23], version 7.3.2. Best tree was estimated by conducting 50 ML searches on 50 random starting trees. Support for internal branches was evaluated by performing 1000 bootstrap replicates. Node support values were mapped on the best tree. Trees were visualized with FigTree v1.3.1 (http://tree.bio.ed.ac.uk/software/figtree/). See supporting material for alignment, tree and sequences.

### Sequence Analysis

All sequences were retrieved from the NCBI (http://www.ncbi.nlm.nih.gov/) or ENSEMBL (http://www.ensembl.org/index.html) databases. Sequencing data deriving from the on campus sequencing core facility (Molecular Neurochemistry, Ruhr University Bochum, Germany) were processed with 4Peaks (Mekentosj, Aalsmeer, The Netherlands) prior to analysis with the ClustalW2 multiple sequence alignment tool (http://www.ebi.ac.uk/Tools/msa/clustalw2). BLAST analysis (BLASTN, BLASTP) was performed using the NCBI or ENSEMBL gateway. Prediction of transmembrane helices and topology of proteins were done using the HMMTOP tool of the Hungarian Academy of Sciences (http://www.enzim.hu/hmmtop/). Asparagine glycosylation sites were predicted using the NetNGlyc 1.0 server (http://www.cbs.dtu.dk/services/NetNGlyc/). Some of the primary sequence analyses were performed using the software tools implemented on the ExPASy website (http://www.expasy.org).

## Results

### Identification and cloning of drPanx1b

**Figure 1:**
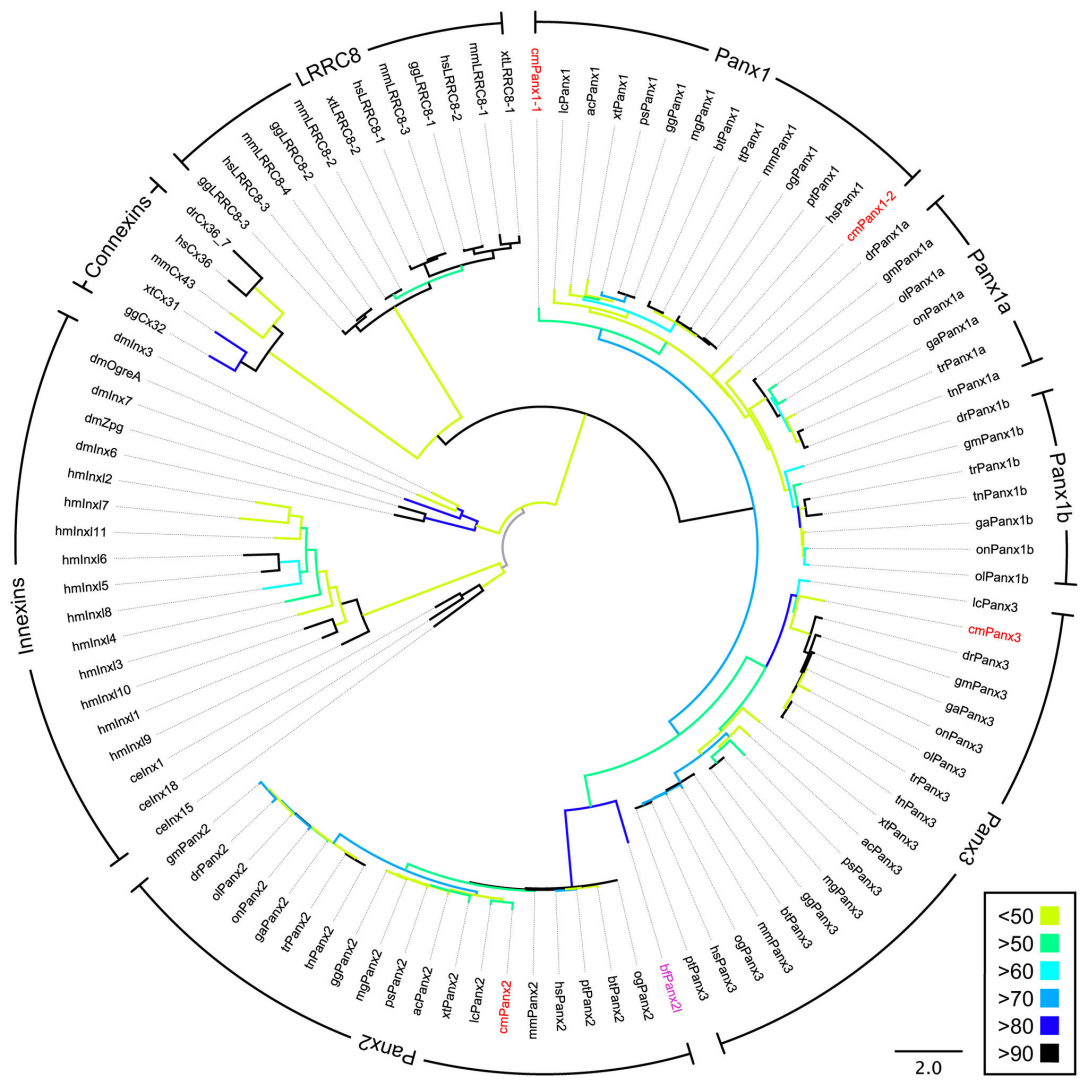
Phylogenetic tree of pannexin protein sequences. Branch colors represent bootstrap values (see right bottom corner). Sequences found in the ghost shark genome are highlighted in red, the sole lancelet sequence found shares similarity with Panx2 sequences (Panx2-like (Panx2l, purple). Zebrafish Panx1a and Panx1b are marked in bold. Tree was rooted to *Hydra* innexin (Inx) sequences. (ac = A*nolis* *carolinensis*; bf = *Branchiostoma floridae*; bt = *Bos taurus*; ce = *Caenorhabditis elegans*; cm = *Callorhinchus milii*; Cx = Connexin; dm = *Drosophila melanogaster*; dr = *Danio rerio*; ga = *Gasterosteus aculeatus*; gg = Gallus gallus; gm = Gadus morhua; hm = *Hydra* *magnipapillata*; hs = Homo sapiens; lc = *Latimeria chalumnae*; LRRC = leucine-rich repeat-containing; mg = *Meleagris gallopavo*; mm = Mus musculus; og = *Otolemur garnettii*; ol = *Oryzias latipes*; on = *Oreochromis niloticus*; Panx = Pannexin; ps = *Pelodiscus sinensis*; pt = Pan troglodytes; tn = *Tetraodon nigroviridis*; tr = *Takifugu rubripes*; tt = *Taeniopygia guttata*; xt = *Xenopus tropicalis*).

With the use of public databases, a second pannexin sequence, termed drPanx1b, (GI: 153791522) was identified in the zebrafish (*Danio rerio*) genome. Phylogenetic analysis revealed similar sequences in the genomes of all *Teleostei* investigated, a fact that is best explained by a third WGD event occurring in the ancestral teleost lineage [14]. In the ghost shark genome, a representative of cartilaginous fish (*Chondrichthyes*), two partial sequences of Panx1 were identified besides Panx2 and Panx3 sequences. In the lancelet genome, representing primitive fish-like chordates, a Panx2-like (Panx2l) sequence was found. All *Hydra* sequences found show more sequence similarities to innexins than to pannexins. Since no sequences were found in the genomes of *Ciona* and *Hydra*, it is very likely that pannexins evolved at a time point when urochordata and chordata separated during evolution. A phylogenetic tree was calculated including all pannexin sequences, selected innexin, connexin and LRRC8 (leucine-rich repeat–containing 8) sequences, the latter being a novel class of putative channel proteins sharing similarities to pannexins (Figure 1; for full tree containing bootstrap values see Figure S1). Sequence identity scores of drPanx1a, drPanx1b and mPanx1, the three Panx1 proteins further investigated in this study, are in the range of 55% - 57%. As sequence alignment between mPanx1, drPanx1a and drPanx1b is depicted in Figure S2 and for drPanx1a and drPanx1b in Figure S3.

The predicted *drpanx1b* gene of 2906 bp contains seven exons with a protein-coding region of 1269 bp, and is located on chromosome 5 at the nucleotide positions 39.439.311-39.454.544. The identity score of the cloned drPanx1b PCR product showed 99.0% identity to its reference sequence (NM_001100030.1, GI: 153791522). A single nucleotide exchange (C712T) was detected resulting in a H238Y amino acid exchange of the 422 amino acid protein. The calculated molecular weight (MW) of the drPanx1b protein is 47.8 kDa. drPanx1b shares the same predicted membrane topology with all known Panx1 proteins. 

### Distinct localization of drPanx1b and drPanx1a in the zebrafish retina

**Figure 2:**
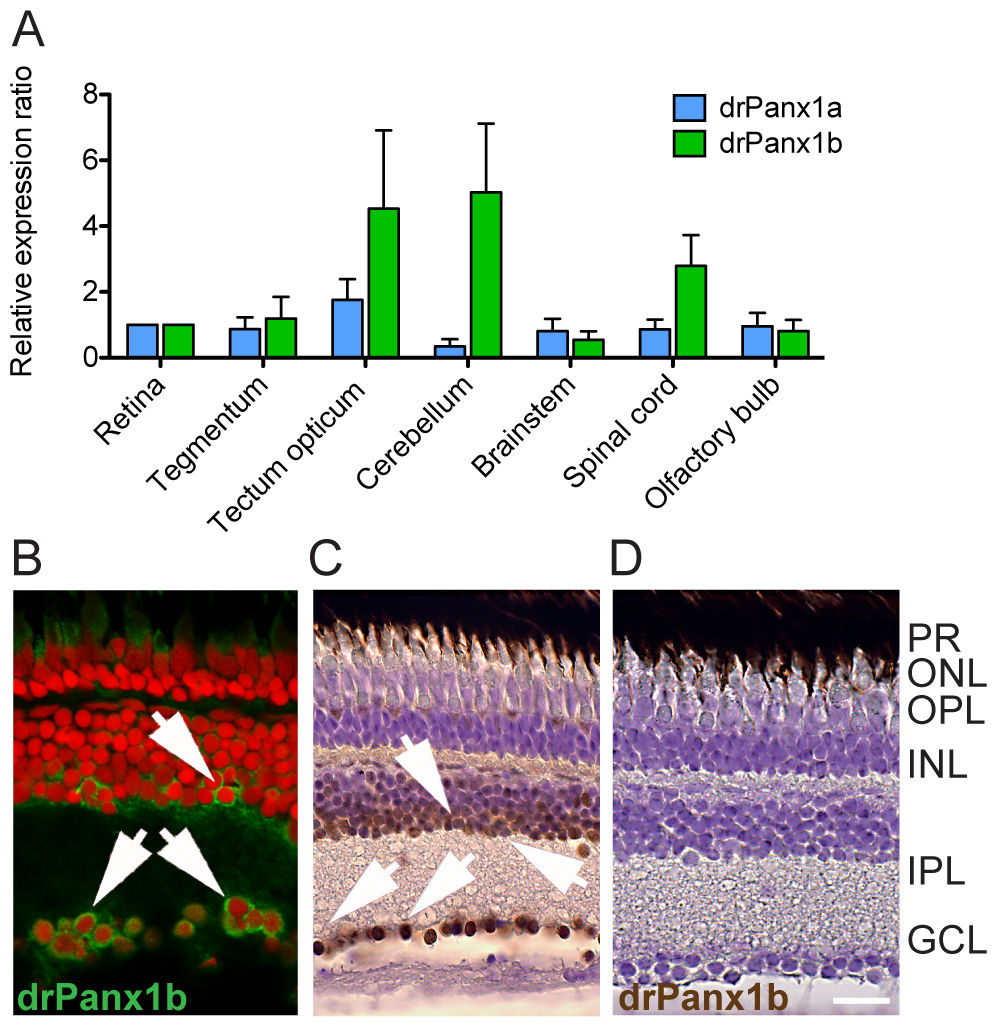
Expression analysis of drPanx1a and drPanx1b in the brain, eye and retina. (**A**) *qRT-PCR analysis of drPanx1 expression*. Total RNA was isolated from several CNS regions of adult zebrafish und used for cDNA synthesis. Retinal drPanx1 expression was normalized to 1. Each bar represents the mean + SEM. (n = 3) (**B**-**D**) *IHC/DAB staining of drPanx1b expression in the retina*. The anti-drPanx1b antibody was used as primary antibody. Cell bodies were stained with propidium iodide (red; B) or cresyl violet (violet; C, D). Arrows point at drPanx1b positive cells in the INL and GCL. (**B**) The primary antibody was visualized by an anti-chicken antibody conjugated with Alexa488. (**C**, **D**) For DAB staining, a biotinylated anti-chicken antibody served as detection antibody. (**D**) Control staining using the immunizing blocking peptide together with the anti-drPanx1b antibody. (Scale bar (B-D) = 10 µm; GCL = ganglion cell layer; INL = inner nuclear layer; IPL = inner plexiform layer; ONL = outer nuclear layer; OPL = outer plexiform layer; PR = photoreceptors).

Expression in the CNS was quantified by qRT-PCR. drPanx1b mRNA expression was found in all major divisions of the adult fish brain (Figure 2A). Relative to the retina, drPanx1b expression levels were most prominent in the cerebellum, tectum opticum and spinal cord. The expression of drPanx1a was lower and less variable in the analyzed CNS tissues. The localization of drPanx1a proteins was previously described in the outer plexiform layer (OPL) in a band like pattern, representing horizontal cells [10]. Western blot analyses proved that the anti-drPanx1a antibody did not recognize drPanx1b (see Figure S4). Although the anti-drPanx1b antibody failed in western blot analyses, anti-drPanx1b immunoreactivity was prominent in the inner nuclear layer (INL) and ganglion cell layer (GCL) (Figure 2B, C). No signal was detected in the inner plexiform layer (IPL), OPL and control experiments with immunizing peptide blocking primary antibodies (Figure 2D) or when primary antibodies were omitted (not shown). The distinct localization of the two Panx1 proteins in the adult fish retina suggests that drPanx1b and drPanx1a could fulfill distinct, but complementary functions *in vivo.*

### Unique glycosylation of drPanx1b affects localization *in vitro*

**Figure 3:**
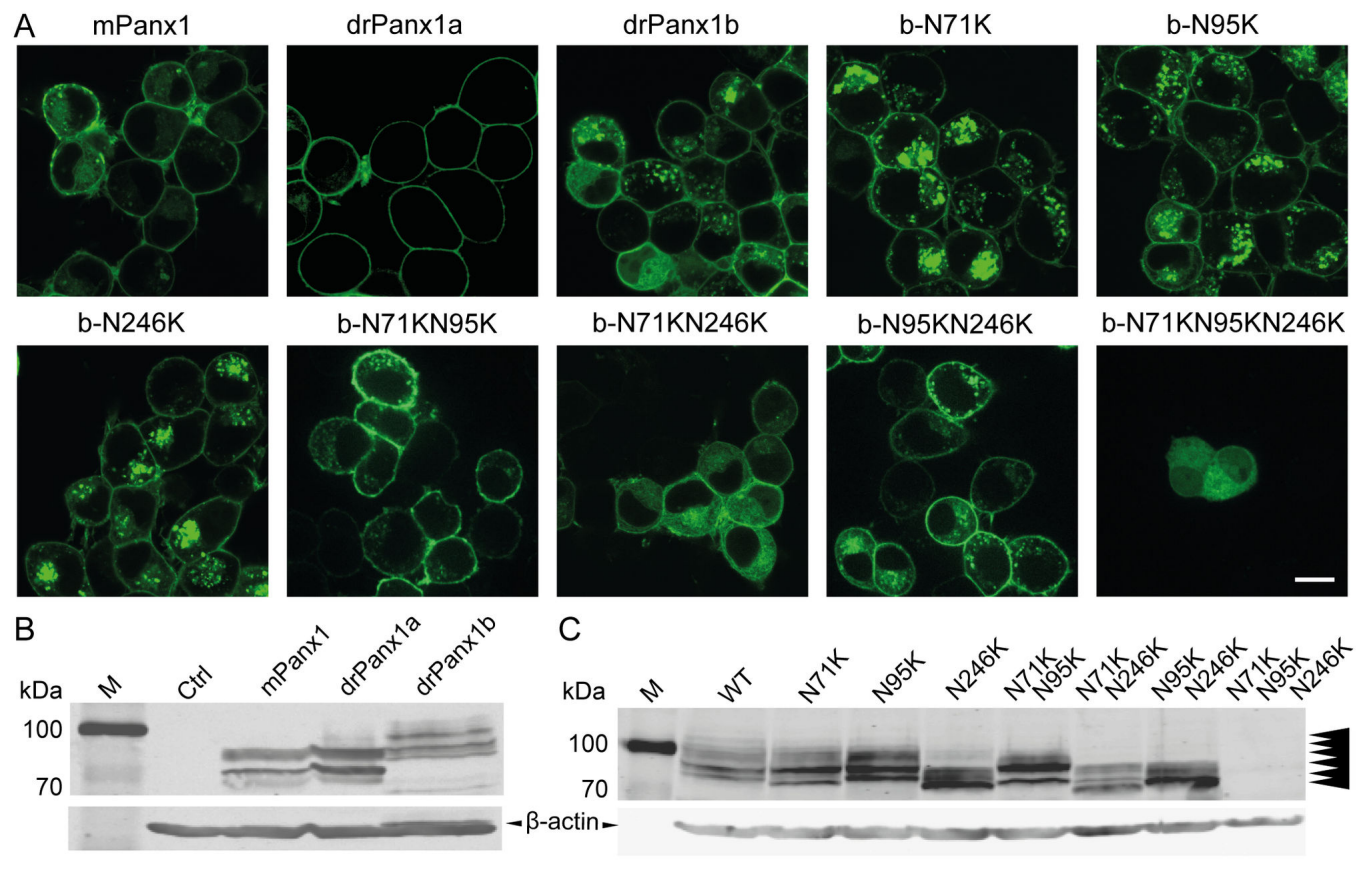
Subcellular localization and western blot analyses of WT and mutant Panx1. (**A**) *Localization of Panx1-EYFP WT and glycosylation-deficient drPanx1b-EYFP mutant proteins in N2a cells*. Protein-derived fluorescence was detected using confocal laser scanning microscopy 48 h post transfection. (Scale bar = 10 µm) (**B**) *Western* *blot* *analyses* *of* *Panx1-EYFP* *WT* *and* (**C**) *glycosylation-deficient* *drPanx1b-EYFP* *mutants*. N2a cells transiently transfected with pEYFP-N1 expression vector constructs were lysed 48 h post transfection. 20 µg total protein were subjected to SDS-PAGE and subsequent western blot analyses using the mouse anti-GFP and anti-mouse IRDye680RD antibodies (B) or rabbit anti-GFP and anti-rabbit IRDye680LT for detection of the Panx1 fusion proteins (C). Mouse anti-β-actin served as a control for equal protein loading and was detected using anti-mouse IRDye680RD (B) or anti-mouse IRDye800CW (C). Arrowheads in (C) indicate the six drPanx1b bands.

The subcellular localization of Panx1 proteins was analyzed in transiently transfected mouse Neuroblastoma 2a (N2a) cells expressing C-terminally EYFP-tagged Panx1 fusion proteins, containing the full coding regions of either drPanx1b (aa 1-422), drPanx1a (aa 1-417) or mPanx1 (aa 1-426). mPanx1 is the best characterized pannexin protein and served as reference protein for subsequent experiments. In N2a cells, drPanx1b, mPanx1, and especially drPanx1a are prominently localized in the plasma membrane 48 h post transfection (Figure 3A). drPanx1b and mPanx1 were located in the plasma membrane and perinuclear region, whereas drPanx1a was mainly found in the cell membrane. Fluorescent aggregates, most pronounced for drPanx1b, potentially reflected rate limiting steps and/or local accumulation during the life cycle of these proteins. Similar to drPanx1a [10], drPanx1b does not show gap junction-like assemblies in the plasma membrane, suggesting that drPanx1b, like mPanx1 and drPanx1a, preferentially forms unopposed membrane channels. The distributions of the three constructs by confocal imaging are consistent with previous reports demonstrating a plasma membrane expression for Panx1 [10,11,24,25].

Since the anti-drPanx1b antibody was unsuitable for western blot, EYFP tagged Panx1 protein expression was used to reveal post-translational modifications using an antibody specific for EYFP detection. Consistent with previous studies reporting N-glycosylation sites in EL2 at N254 for mPanx1 or N246 for drPanx1a [10,11,25–27], we confirmed the Panx1-specific band pattern corresponding to the unmodified Gly0 core proteins, high mannose Gly1 species and complex glycosylated Gly2 species (Figure 3B). The pattern for drPanx1b was more complex, showing motilities different from the calculated MW of 76.6 kDa for the fusion protein, indicating more extensive post-translational modifications.

For drPanx1b, the NetNGlyc 1.0 server predicted putative N-glycosylation sites in EL1 (NFTM, aa position 71-74; NETY, aa position 95-98) and EL2 (NQSE, aa position 246-249). Using site-directed mutagenesis, the aspargines (N) was replaced with lysines (K) at positions 71, 95 and 246 to test for glycosylation deficiency of the mutant drPanx1b-N71K, -N95K and -N246K proteins. In addition, double (drPanx1b-N71KN95K, -N71KN246K and N95KN246K) and triple (drPanx1-N71KN95KN246K) mutants were generated. The qualitative analysis revealed no obvious differences in the subcellular localization of WT and N71K, N95K, N246K, N71KN95K and N95KN246K mutants (Figure 3A). In contrast, the N71KN246K double and N71KN95KN246K triple mutants appear to be retained in cytoplasmic compartments, suggesting that these mutants are not efficiently transported or inserted in the plasma membrane due to improper glycosylation. Further, the transfection efficiency of the triple mutant is low, with a diffuse EYFP signal found also in the cell nucleus, which we consider to indicate a pronounced degradation of this mutant.

Western blot analysis showed at least six bands for drPanx1b WT (arrowheads, Figure 3C). The two lowest bands are most prominent with MW above 76 kDa, where non-glycosylated drPanx1b is expected. The N71K and N246K mutations clearly shift the lowest band to lower MW, which is most prominent in the N71KN246K double mutant. Further, bands above 100 kDa are reduced in the N71K and N95K and absent in the N246K and all double mutants. In contrast to the drPanx1a-N246K mutant showing only one clear band [10], drPanx1b-N246K still shows several prominent bands, indicating that glycosylation (or other types of post-translational modifications) takes place at other site(s). The N95K mutation does not obviously change the band pattern, no matter whether the single mutant is compared to the WT or the double mutants N71KN95K or N95KN246K to the corresponding single mutants N71K or N246K. Interestingly, generation of the N71KN95KN246K triple mutant results in a drastic reduction of protein expression, making it undetectable on the western blot. In summary, the data clearly identifies N71 and N246 as glycosylation sites, but since no prominent differences where found for the N95 mutant(s) it is hard to determine whether this site is glycosylated or not. However, since all double mutants are well expressed, but not the triple mutant, this indicates that N95 seems to be somehow important. With respect to changes in the complexity of the glycosylation pattern, these results are consistent with the subcellular localization where the drPanx1b-N single mutants are efficiently inserted into the cell membrane, whereas the N71KN246K double and N71KN95KN246K triple mutants are mainly retained in the cytoplasm. 

### drPanx1b and drPanx1a channels show Ca^2+^-dependence and pH sensitivity

**Figure 4:**
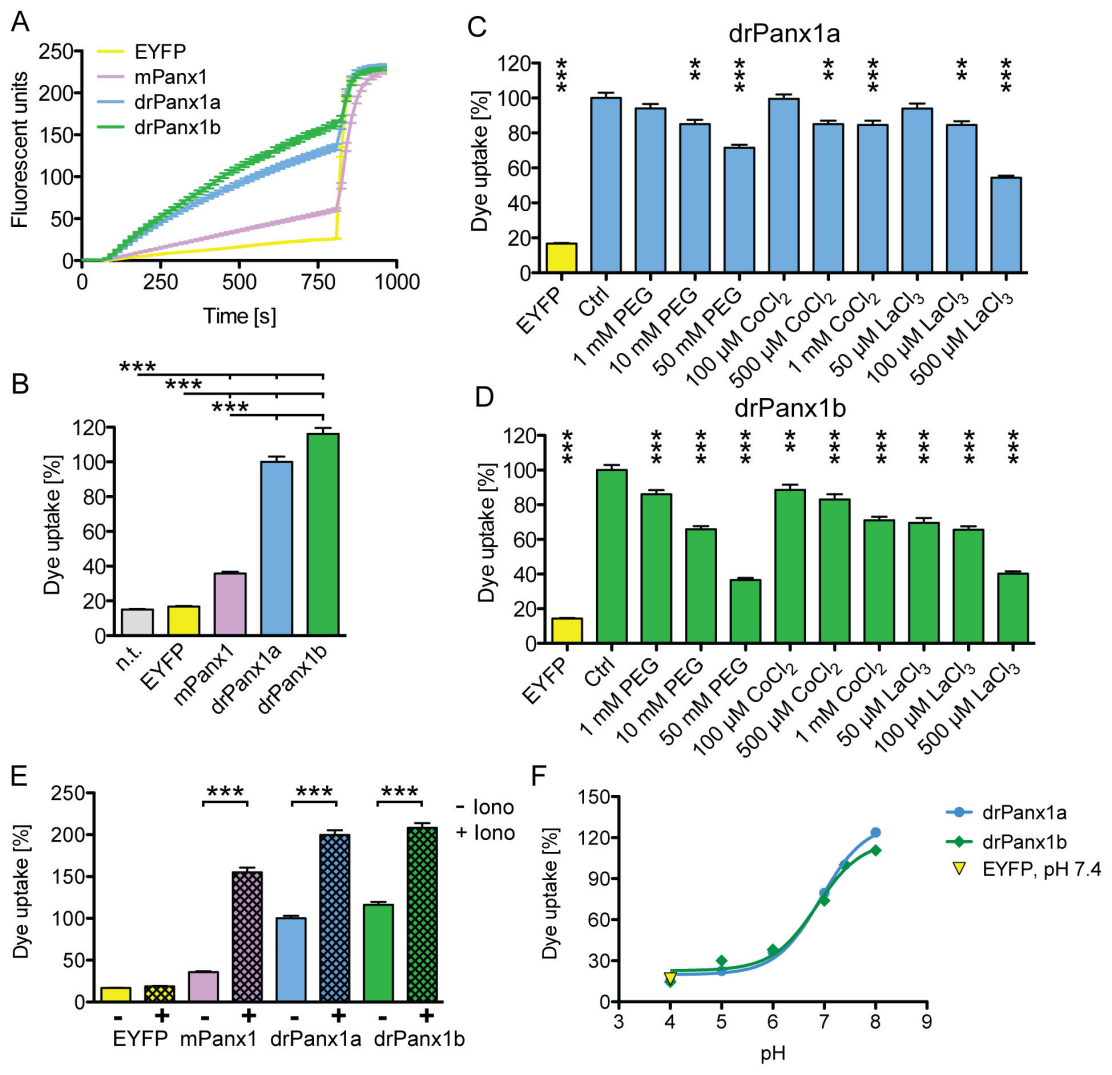
Calcium and pH dependence dye uptake of N2a cells expressing Panx1 proteins. N2a cells expressing EYFP or EYFP-tagged mPanx1, drPanx1a or drPanx1b were used for dye uptake assays 48 h post transient transfection. Ethidium bromide (EtBr) fluorescence was compared 5 min after EtBr (20 µM) application. Each bar represents the mean + SEM of 135 analyzed cells. (**A**) *Time* *course* *and* (**B**) *statistical* *analyses* *of* *the* *dye* *uptake* *of* *EYFP* or *Panx1* *expressing* *N2a* *cells*. All values were normalized to the averaged EtBr uptake of drPanx1a expressing cells, which was set to 100%. (p<0.001 = ***) (**C**, **D**) *Pharmacological reduction of dye uptake in (C) drPanx1a and (D) drPanx1b transfectants*. Multiple well-described connexin hemichannel and Panx1 blockers were applied together with EtBr to the external solution. All values were normalized and compared to the control condition of drPanx1a (C) or drPanx1b (D) expressing cells (Ctrl), which was set to 100%. (**E**) *Elevation of dye uptake mediated by Ca^2+^/ionomycin stimulation in EYFP or Panx1 expressing N2a cells*. Ionomycin (Iono) was applied together with EtBr. All values were normalized to the averaged EtBr uptake of drPanx1a expressing cells, which was set to 100%. The dye uptake was compared between ionomycin stimulated (+) cells and the respective control condition (-). (**F**) *Influence of the extracellular pH on dye uptake in drPanx1 transfected N2a cells*. All values were normalized to the control condition at pH 7.4 of either drPanx1a (blue) or drPanx1b (green) expressing cells, set to 100%. A sigmoidal curve fit was performed. The EYFP control at pH 7.4 is depicted in yellow. (PEG = PEG1500; n = 135 cells per group; p>0.05 = ns; p<0.05 = *; p<0.01 = **; p<0.001 = ***).

Dye uptake assays demonstrated that drPanx1a operates as unopposed channel under physiological conditions [10,11]. Using dye uptake assays, we compared drPanx1b channel activity to drPanx1a and mPanx1. EYFP expressing and non-transfected (n.t.) cells were used as controls (Figure 4A, B). All values were normalized to the dye uptake of drPanx1a transfectants 5 min after application of EtBr (20 µM), being set to 100% (Figure 4B). No difference was detected between control cells (n.t.: 15.0 ± 0.4%; EYFP: 16.7 ± 0.3%), indicating that dye uptake was not induced by the transient transfection procedure. Compared to EYFP controls, EtBr uptake was significantly increased in mPanx1 (35.7 ± 1.0%, p<0.001), drPanx1a (100.0 ± 3.0%, p<0.001) and drPanx1b (116.2 ± 3.4%, p<0.001) transfectants. Uptake of EtBr in drPanx1a and drPanx1b transfectants was not significantly different. The significant correlation of membrane fluorescence and EtBr uptake (see Figure S5) confirms that dye uptake correlates with pannexin expression. Gap junction and hemichannel blockers polyethylene glycol 1500 (PEG1500) [7], CoCl_2_ [28] and LaCl_3_ [29] were tested to evaluate the capability to block dye uptake in drPanx1a and drPanx1b expressing N2a cells. All compounds significantly inhibited dye uptake in a concentration dependent manner (Figure 4C, D). Maximum inhibition of dye uptake was: PEG1500 50 mM: drPanx1a 28.4 ± 1.7%, p<0.0001; drPanx1b: 63.4 ± 1.2%, p<0.0001; CoCl_2_ 1 mM: drPanx1a: 15.5 ± 2.5%, p=0.004; drPanx1b: 29.0 ± 2.0%, p<0.0001; LaCl_3_ 500 µM: drPanx1a: 45.6 ± 1.1%, p<0.0001; drPanx1b: 59.8 ± 1.3%, p<0.0001). In all cases, drPanx1b transfectants were more sensitive to blockers than cells expressing drPanx1a. Taken together, these data show that fish Panx1 proteins form active, pharmacosensitive channels mediating dye uptake under resting conditions.

Elevation of cytosolic Ca^2+^ is a critical factor modulating human Panx1 channel opening [6]. To investigate if fish and mouse Panx1 share this property, dye uptake assays were performed in the presence of 10 µM of the Ca^2+^ ionophore ionomycin (iono) (Figure 4E). In vector-only controls, Ca^2+^/iono stimulation did not significantly increase EtBr uptake (11.9 ± 2.9%), demonstrating that ionomycin itself does not cause uptake of EtBr. Exogenous Panx1 expression and Ca^2+^/iono stimulation enhanced EtBr uptake highly significantly. Relative to non-stimulated drPanx1a (set to 100%), mPanx1 transfectants displayed the highest increase (334.5 ± 15.7%, p<0.001), followed by drPanx1a (99.6 ± 5.7%, p<0.001) and drPanx1b (79.3 ± 4.7%, p<0.001). In absolute terms, dye uptake of drPanx1a (199.6 ± 5.7%; p<0.05) and drPanx1b (208.3 ± 5.5%; p<0.01) cells remained higher than uptake of mPanx1 transfectants after stimulation (155.1 ± 5.6%). We conclude that Panx1 channels from different species respond to changes in intracellular calcium with different efficacies.

Connexin hemichannel activity is regulated by pH changes [30] and cytoplasmic acidification closes human Panx1 [6] and rat Panx2 [31] channels. Here, manipulating the pH through application of extracellular solutions in a pH range from pH 4 to pH 8 was used to test the pH sensitivity of drPanx1 channels. Compared to control levels at pH 7.4 (set to 100%), extracellular acidification decreased EtBr uptake (Figure 4F) (drPanx1a/drPanx1b: pH 7: -20.4 ± 1.8%, p>0.05/-26.1 ± 1.8%, p<0.01; pH 6: -63.9 ± 0.9%, p<0.001/-61.8 ± 0.9%, p<0.001; pH 5: -77.5 ± 0.6%, p<0.001/-70.0 ± 0.9%, p<0.001; pH 4: -84.8 ± 0.4%, p<0.001/-85.4 ± 0.4%, p<0.001), whereas extracellular alkalization increased channel activity (drPanx1a/drPanx1b: pH 8: +23.8 ± 2.8%, p<0.05/+10.7 ± 2.4%, p>0.05). At pH 4, no differences between drPanx1 transfectants and vector-only controls at pH 7.4 were found. The sigmoidal curve fit revealed following half maximal values: drPanx1a pH 6.93 and drPanx1b pH 6.86. The results indicate that acidic extracellular conditions promote closure of drPanx1a and drPanx1b channels, whereas a moderate alkalization opens these channels. In summary, drPanx1 channels are active at resting conditions, modulated by an increase of cytosolic Ca^2+^ and respond to pH changes within physiologic ranges found in different tissues. 

### drPanx1b and drPanx1a channels can be inhibited by ATP and BBG

**Figure 5:**
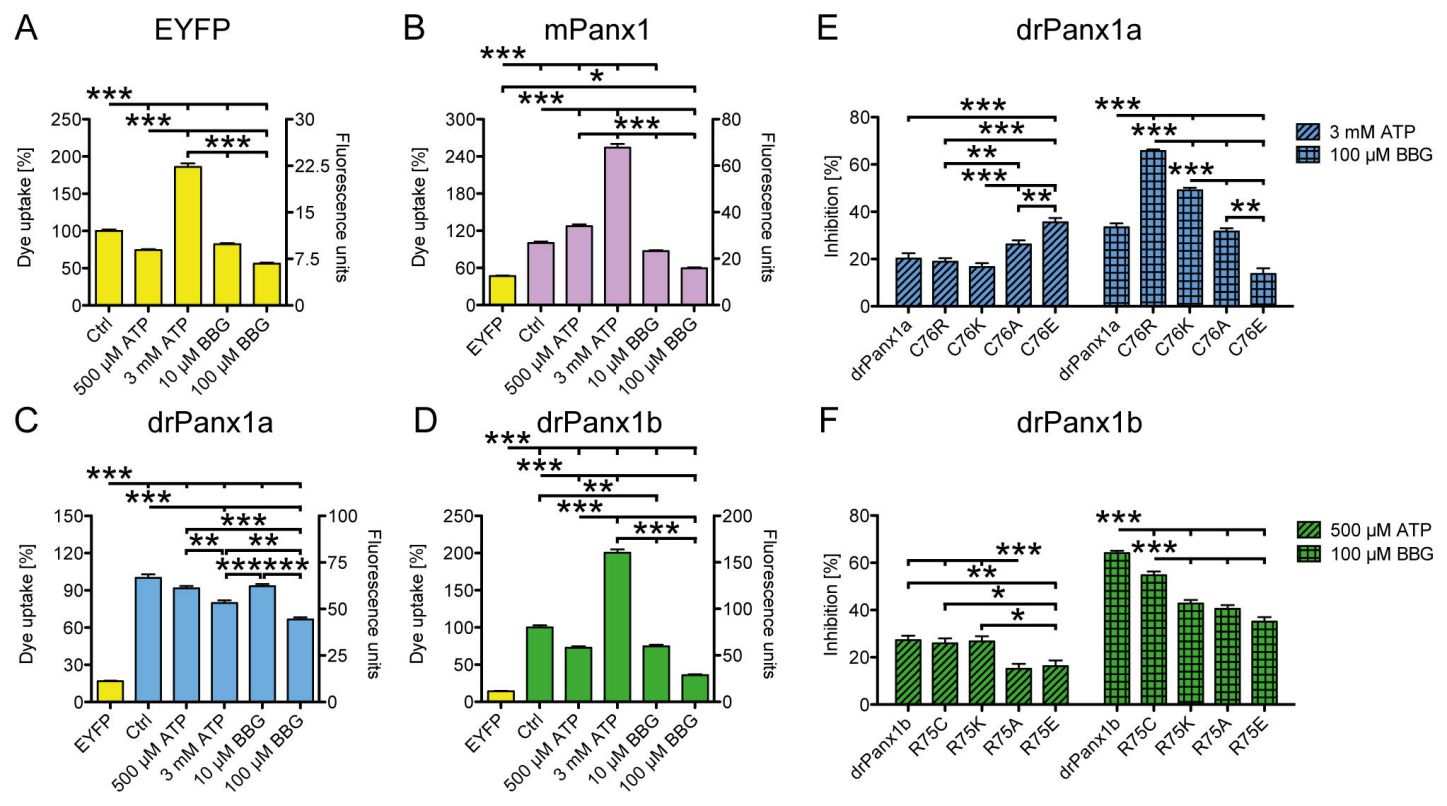
Influence of ATP and BBG on dye uptake of EYFP/Panx1 WT mutant expressing N2a cells. N2a cells expressing (A-D) EYFP or EYFP-tagged mPanx1, drPanx1a, drPanx1b or (E, F) drPanx1a-C76R, -C76K, -C76A, -C76E or drPanx1b-R75C, - R75K, -R75A, -R75E mutants were used for dye uptake assays 48 h post transient transfection. ATP or BBG were applied together with EtBr (20 µM). Each bar represents the mean of EtBr fluorescence + SEM of 135 cells analyzed 5 min after EtBr/compound application. All values were normalized to the respective control condition (Ctrl), which was set to 100%. (p<0.001 = ***). (**A**-**D**) *Influences of ATP and BBG on dye uptake in EYFP or Panx1 expressing N2a cells*. The total EtBr uptake in relative fluorescence units is depicted on the right y-axis. In (B)-(D), the basal dye uptake of EYFP expressing cells is depicted as a reference. (**E**,**F**) *Cross-comparison of the ATP- and BBG-mediated reduction of dye uptake in (E) drPanx1a WT and -C76 mutant as well as in (F) drPanx1b WT and -R75 mutant expressing N2a cells*. The respective control condition set to 100% is not depicted. (n = 135 cells per group; p<0.05 = *; p<0.01 = **; p<0.001 = ***).

In mammals, Panx1 is considered as a major ATP release channel (e.g. [32]), being modulated by extracellular ATP (e.g. [6]). To test whether this property is also true for non-mammalian Panx1, dye uptake assays were used to assess Panx1 function when applying ATP or the non-competitive antagonist of P2X_7_ receptors brilliant blue G (BBG), previously shown to block murine Panx1 [8] (Figure 5A-D). Application of 500 µM or 3 mM ATP to mPanx1 transfected cells resulted in significantly enhanced EtBr uptake (500 µM: +27.4 ± 2.8%, p<0.001; 3 mM: +154.0 ± 6.2%; p<0.001; Figure 5B). In contrast, application of 500 µM ATP to EYFP (Figure 5A) or drPanx1b (Figure 5D) expressing cells led to a significant reduction (EYFP: -25.7 ± 1.3%, p<0.001; drPanx1b: -27.3 ± 1.9%, p<0.001), whereas 3 mM ATP caused enhanced EtBr uptake (EYFP: +86.0 ± 4.7%, p<0.001; drPanx1b: +100.4 ± 4.5%; p<0.001). Interestingly, for drPanx1a transfectants we could only observe ATP-mediated inhibition (Figure 5C) that was significant when 3 mM ATP were used (500 µM ATP: -8.3 ± 1.9%, p>0.05; 3 mM ATP: -20.2 ± 2.2%; p<0.001). The application of BBG was inhibitory in all cases with a maximum inhibition found for drPanx1b at 100 µM (drPanx1b: -64.1 ± 1.0%, p<0.001 > mPanx1: -40.6 ± 1.4%, p<0.001 > drPanx1a: -33.4 ± 1.7%, p<0.001 > EYFP: -43.9 ± 1.3%, p<0.001).

Panx1 channels contain no affirmed ATP binding sequence, but Qiu and Dahl (2009, [8]) identified a critical arginine residue in the EL1 (R75) as being involved in ATP binding. Interestingly, this residue is conserved in drPanx1b, but not in drPanx1a (GI: 41055475, GI: 182892107), which contains a third cysteine at the equivalent position (C76). Since this variation was found in all clones originally identified in our group [10,11], we tested whether this natural variant altered ATP binding properties. As shown above, drPanx1b is more sensitive to ATP or BBG than drPanx1a. To clarify the role of drPanx1a-C76 or drPanx1b-R75 in this response, we replaced these amino acids with R/K/A/E or C/K/A/E, respectively. Some of these mutations caused distinct subcellular redistribution and altered dye uptake properties (see Figure S6). The drPanx1a-C76R, -C76K and -C76A mutants appeared more retained in cytoplasmic compartments, suggesting impaired cell surface trafficking as a cause for the decreased EtBr uptake. In contrast, comparing drPanx1a to the -C76E mutant and drPanx1b to its R75 mutants, no obvious differences in subcellular localization, western blot and dye uptake were detected.

Next, dye uptake assays demonstrated that either application of 3 mM ATP for drPanx1a and 500 µM ATP for drPanx1b variants as well as 100 µM BBG for both resulted in a significantly decreased EtBr uptake (p<0.01 - p<0.001) when compared to control conditions (not depicted; Figure 5E, F). Interestingly, all drPanx1b and drPanx1a variants, except for drPanx1a-C76E, were more sensitive to BBG than to ATP. Like Qiu and Dahl (2009), we detected different sensitivities to ATP or BBG depending on the respective aa at position 76/75, with the following rank orders from higher to lower sensitivity: ATP: drPanx1a-C76E > C76A ≥ WT ≥ C76R ≥ C76; drPanx1b WT ≥ R75K ≥ R75C > R75E ≥ R75A; BBG: drPanx1a-C76R > C76K > WT ≥ C76A > C76E; drPanx1b WT > R75C > R75K ≥ R75A ≥ R75E. Thus, we conclude that ATP-mediated modulation of Panx1 channel activity and the critical role of EL1 in ATP binding is an evolutionary conserved feature between mammals and non-mammals, pointing at the physiological relevance of this function.

### drPanx1b forms voltage-gated channel with distinct gating properties

**Figure 6:**
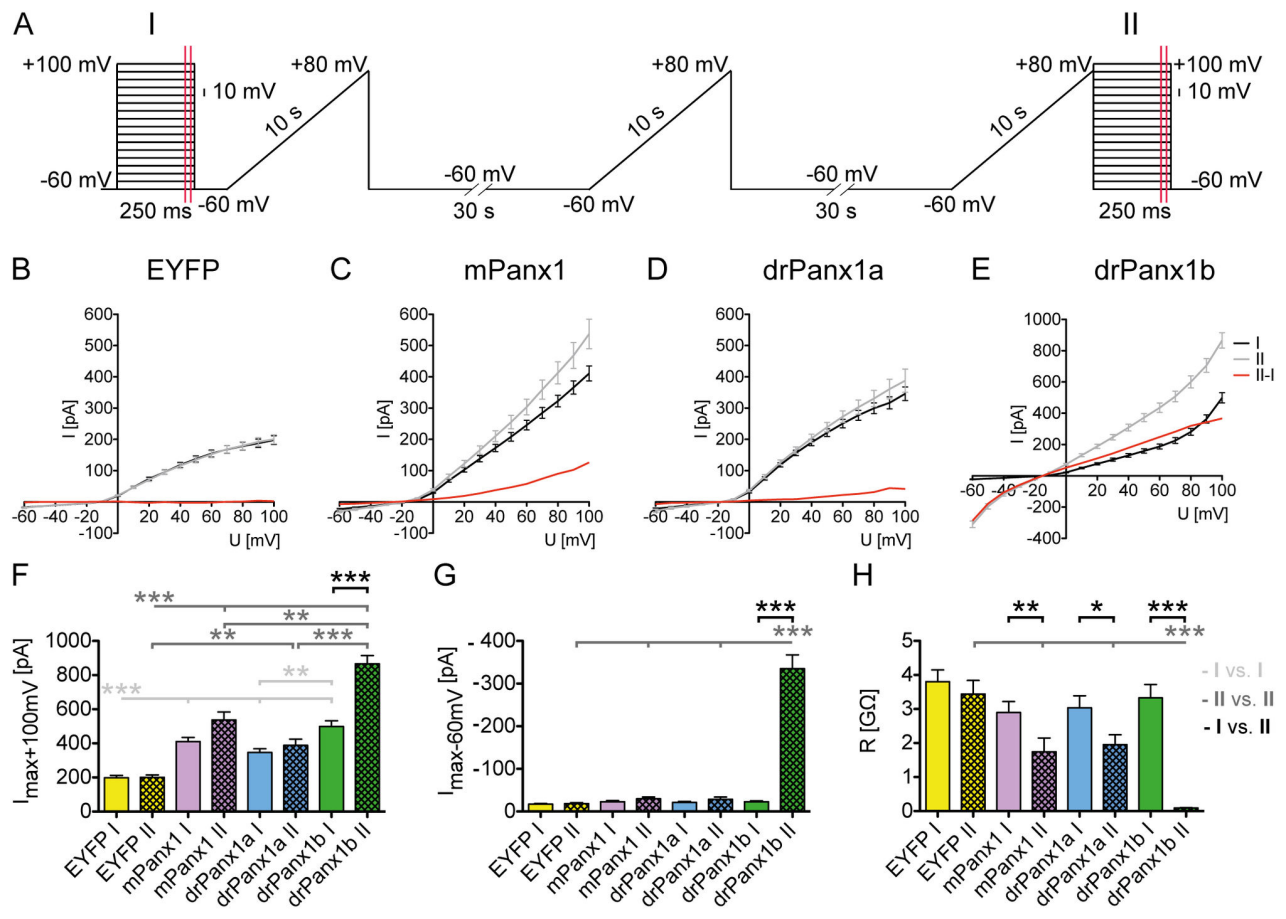
Outline of the preconditioning protocol and I/V relation before/after preconditioning of EYFP/Panx1 expressing N2a cells. (**A**) *Scheme of the preconditioning protocol*. Depolarizing voltage steps were applied before (**I**) and after (II) three preconditioning voltage ramps. For statistical analyses of the I/V relation, the current response during 10 ms at the end of the voltage steps (depicted by the red lines) was averaged. N2a cells expressing EYFP or EYFP-tagged mPanx1, drPanx1a or drPanx1b were used for whole-cell patch clamp recordings in the voltage clamp mode 48 h post transfection. (**B**-**E**) *I/V relations calculated from the voltage step protocol **I** and **II***. The difference of the I/V relations from **I** and **II** (red line) was calculated by subtraction of the membrane currents of **I** from **II**. Values are mean ± SEM. (**F**) *Maximum* *current* *amplitudes* *I*_*max*_ *at +100 mV*, (**G**) *maximum* *currents* *amplitudes* *at -60 mV* *and* (**H**) *input* *resistance* *R* *at -60* *mV* *calculated* *from* ***I*** and ***II***. Values were calculated from (B-E). Each bar represents the mean + SEM. For statistical analyses between the values of the different groups obtained as response to the first (depicted in light grey) or second (depicted in dark grey) depolarizing voltage ramps, the Kruskal-Wallis test followed by a Dunn’s Multiple comparison post test was performed. For comparison between the values obtained for **I** and **II** within one transfectant group (depicted in black), the Mann-Whitney test was performed. (n of I/II: EYFP: n  = 45/42; mPanx1: n = 28/21; drPanx1a: n = 37/31; drPanx1b: n = 40/32; p<0.05 = *; p<0.01 = **; p<0.001 = ***).

Whole cell patch clamp recordings of transfected N2a cells were used to analyze the electrophysiological properties of Panx1 channels. Using ion gradient conditions that mimic physiological conditions, a simple voltage step paradigm like I (Figure 6A) opened only a limited number of channels in trial experiments. Since we were interested to promote robust Panx1 channel opening and closure, a combined depolarizing holding voltage step and long ramp preconditioning paradigm (modified from [33] was applied. Previous studies from our group let us chose three ramps to analyze whether a preconditioning effect was long lasting. Here, we compared the Panx1 channel activity before (I) and after (II) voltage driven channel activation (examples of original traces are depicted in Figure S7) and calculated I/V relation before (I) and after (II) preconditioning (Figure 6B-E). These experiments revealed that especially drPanx1b transfectants show strongly increased currents in response to negative and positive voltage steps applied directly after a depolarizing voltage ramp. Only for drPanx1b transfectants the currents increased significantly at +100 mV (Figure 6F) and at -60 mV (Figure 6G). Figure 6E shows the difference between the IV-relations prior and following preconditioning (red line in Figure 6E) and illustrates this point. In general, prolonged depolarization of the membrane potential seems to activate drPanx1b channels more than mPanx1 or drPanx1a channels. Expression of tag-less drPanx1b using pIRES2-mRFP1-drPanx1b revealed only a minor impact of the EYFP-tag, demonstrating that the distinct properties of drPanx1b are channel specific (see Figure S8). Compared to EYFP-controls, mPanx1 and drPanx1a display also significantly increased maximum responses at +100 mV (Figure 6F). In contrast to drPanx1b, both mPanx1 and drPanx1a transfectants lacked a pronounced preconditioning effect regarding significantly enhanced current amplitudes. Nevertheless, in all cases of exogenous Panx1 expression the input resistance R at -60 mV decreased significantly after preconditioning (Figure 6H).

**Figure 7:**
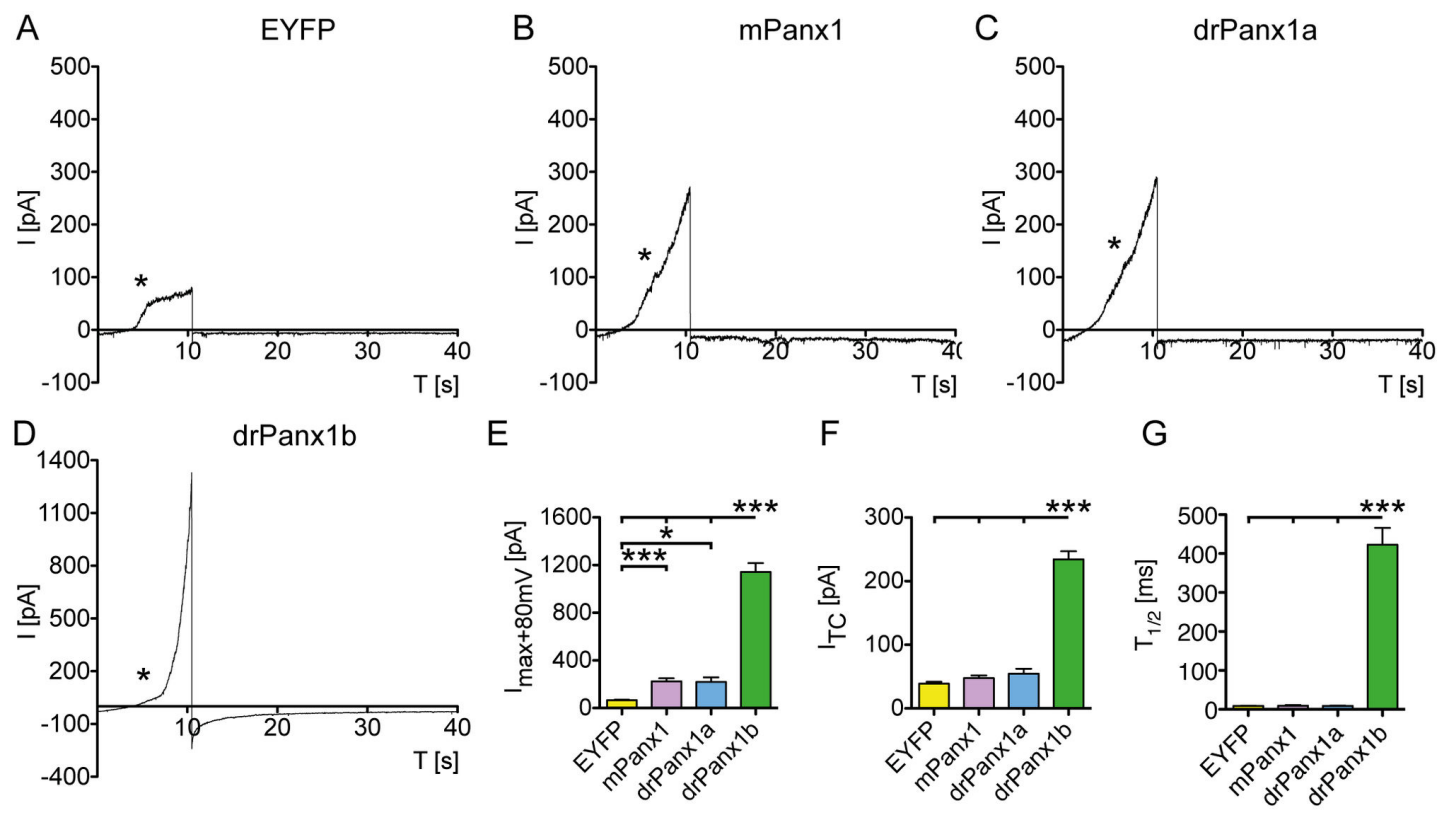
Analyses of membrane currents elicited by depolarizing voltage ramps in EYFP/Panx1 expressing N2a cells. N2a cells expressing EYFP or EYFP-tagged mPanx1, drPanx1a or drPanx1b were used for whole-cell patch clamp recordings in the voltage clamp mode 48 h post transient transfection. Current responses to consecutive depolarizing voltage ramps from -60 mV to +80 mV were recorded within the preconditioning paradigm. (**A**-**D**) *Example traces of the current response elicited by 10 s depolarizing voltage ramps in (A) EYFP, (B) mPanx1, (C) drPanx1 and (D) drPanx1b expressing N2a cells*. Asterisks mark the contribution of outward rectifying currents. This causes a decline in the slopes at membrane potentials between +10 mV and +20 mV in all groups, which is barely visible in drPanx1b transfectants. (**E**) *Maximum current amplitudes I_max_ recorded at +80 mV*. (**F**) *Tail* *current* *amplitudes* *I*_*TC*_ *evoked* *after* *rapid* *hyperpolarization* *from +80 mV* *to -60mV* *after* *the* *first* *depolarizing* *voltage* *ramp* *and* (**G**) *time* *at* *which* *the* *tail* *current* *amplitudes* *decreased* *to 50% of its initial value, T*_*1/2*_ *, of* *the* *repolarization* *current*. All values in (E-G) were calculated from the averaged current responses to the first voltage ramp within the preconditioning paradigm. Each bar represents the mean + SEM. (EYFP: n = 35; mPanx1: n = 28; drPanx1a: n = 24; drPanx1b: n = 36; p<0.05 = *; p<0.01 = **; p<0.001 = ***).

Next, we analyzed the current response elicited by the depolarizing voltage ramps. For this purpose, Panx1 gating kinetics were analyzed using data from the first ramp, since we found no significant differences between the three ramps (not shown). Example traces are shown in Figure 7A-D. The membrane current responses and repolarization currents were comparable between mPanx1 and drPanx1a transfectants, whereas the membrane current amplitudes in drPanx1b transfectants were largely increased. The statistical analyses of the maximum current amplitudes at +80 mV underline these results, with drPanx1b differing from all other transfectants in a highly significant manner (Figure 7E). However, compared to EYFP-controls, mPanx1 and drPanx1a also showed significantly increased maximum responses (I_max_+80mV [pA]: EYFP: 65.4 ± 5.1; mPanx1: 224.9 ± 24.6; drPanx1a: 219.5 ± 38.1; drPanx1b: 1143 ± 74.5). Evidence for high conductance of drPanx1b was also given by the repolarization characteristics. Rapid hyperpolarization from +80 to -60 mV at the cessation of the first ramp lead to significantly larger tail current amplitudes (I_TC_) in drPanx1b transfectants (p<0.001; Figure 7D, F; I_TC_ [pA]: EYFP: 38.9 ± 2.9; mPanx1: 47.6 ± 4.4; drPanx1a: 54.6 ± 7.8; drPanx1b: 234.1 ± 12.8). This result was accompanied by a significantly increased repolarization time constant, T_1/2_ (time needed for a decrease of the tail current amplitude to 50% of the initial value; p<0.001; Figure 7G; T_1/2_ [pA]: EYFP: 8.8 ± 0.5; mPanx1: 9.4 ± 1.7; drPanx1a: 9.0 ± 1.0; drPanx1b: 422.9 ± 43.2). Experiments with N2a cells expressing untagged drPanx1b excluded the possibility that the EYFP tag accounted for the observed differences (see Figure S9). Compared to EYFP controls, cells transfected with mPanx1 or drPanx1a did not reveal significantly increased tail current amplitudes or repolarization time constants. 

### drPanx1b channels exhibit large single channel conductance with multiple subconductance states

**Figure 8:**
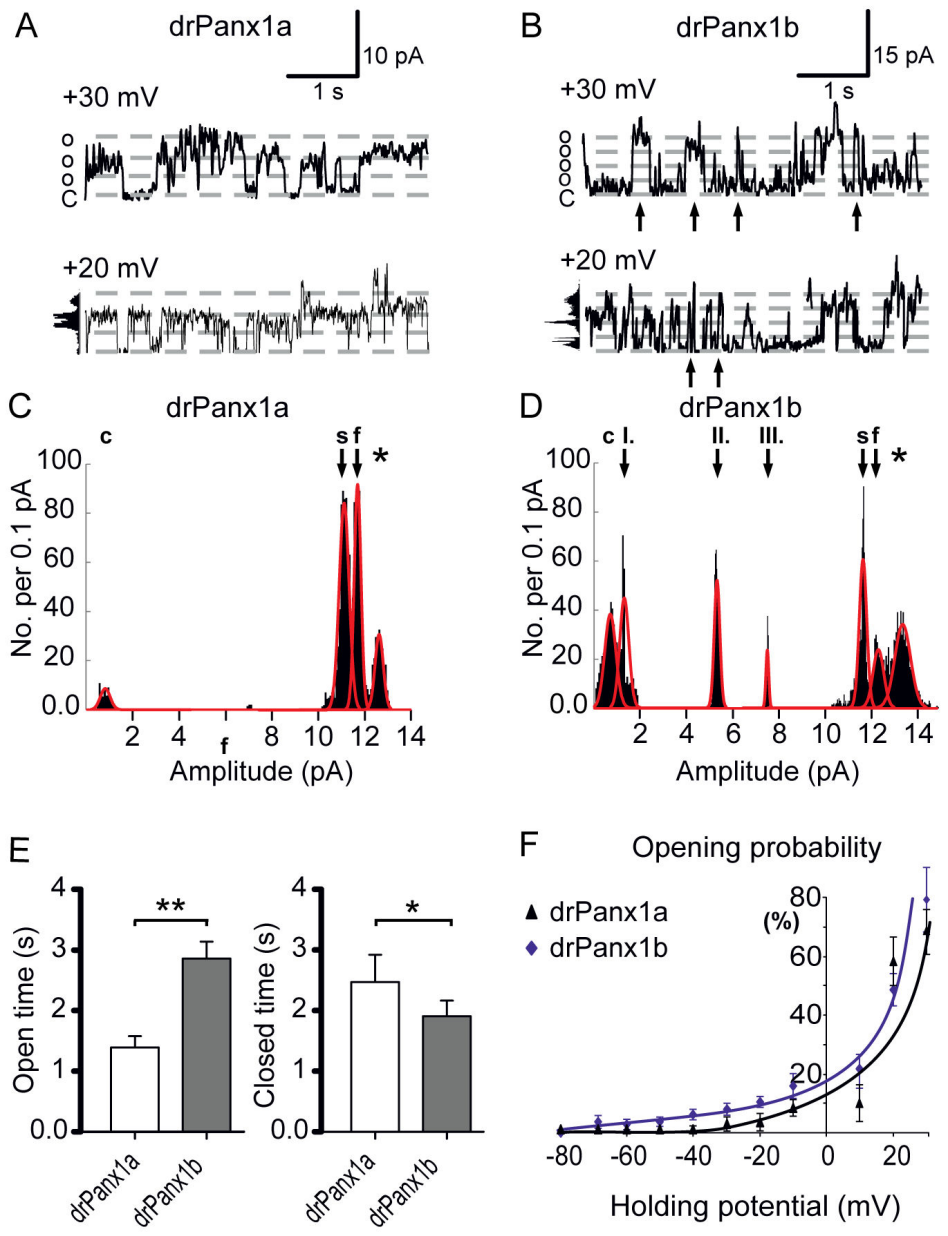
Single channel recordings of drPanx1a and drPanx1b. N2a cells expressing EYFP-tagged drPanx1a or drPanx1b were used for single channel recordings 48 h post transient transfection. (**A**,**B**) *Single channel recordings of drPanx1 channels from outside-out patches at +30 mV*. To elicit channel activity, membrane fragments were processed through depolarizing holding potential steps of (140 s duration; range -60 to +30 mV; increment 10 mV). Both channels display frequent channel activation at positive holding potentials. Arrows in B indicate multiple short-term events. (**C**,**D**) *Subconductance states of drPanx1 channels from the full 140 s of recording at +30 mV*. Intermediate and full open conductance states of the channel are indicated by arrowheads. The full open condition of endogenous mouse Panx1 is labeled by asterisks and was also present in EYFP- and non-transfected controls (data not shown) (c = closed; s = substate; f = fully opened). (**E**) *Comparison of the cumulative full open and closed times of drPanx1 channels*. Data were calculated from times for the fully opened and closed state averaged over five total recording periods of 10 s of each membrane fragment at +30 mV. (E left), comparison of the mean times for the fully opened (left) and closed state (right) for five total recording periods of 10 s at +30 mV. (**F**) Voltage dependent opening probability of drPanx1 channels. (drPanx1a n = 7; drPanx1b n = 10; p<0.05 = *; p<0.01 = **).

Excised outside-out patch clamp recordings in the voltage clamp mode revealed distinct differences and similarities between drPanx1a and drPanx1b transfected N2a cells under physiologic ion gradient conditions. Depolarization elicited inward currents of large single channel conductance in the fully opened state (Figure 8A, B characterizes both channels). The mean single unit conductance of 498 ± 19.5 pS (n = 10) for the fully opened state for five total recording periods of 10 s at -30 mV was significantly higher in drPanx1b transfectants compared to drPanx1a (340 ± 10.8 pS; p < 0.005; (n = 7). 

All point histograms (Figure 8C, D) of the entire 140 s recording period of the excised membrane fragments (samples shown in Figure 8A, B) confirmed the results of previous studies of Prochnow et al. 2009a, 2009b under *in vivo* like solution conditions: aside from the closed (c) and full open (f) condition, drPanx1a transfected N2a cell derived membrane fragments revealed one characteristic subconductance (arrow) state (Figure 8C, mean state amplitude: 11.2 ± 0.1 pA; n = 7). In drPanx1b transfected cells, this pattern was reiterated, including the subconductance state (s) observed for drPanx1a (Figure 8D, mean state amplitude of 11.9 ± 0.6 pA; n = 10). Three additional intermediate conductance states (I. - III.) were detected for drPanx1b, with mean state amplitudes of I. 1.5 ± 0.5 pA, II. 5.2 ± 0.03 pA and III. 7.6 ± 0.02 pA (indexed in Figure 8D). It has to be mentioned that N2a cell membranes revealed a high conductance state at ~13 pA (labeled by asterisks in Figure 8C, D), independent from the transfection mode, which may be caused by endogenous mouse Panx1 channels and was also found in EYFP transfected and non-transfected native cells (data not shown).

 The cumulative times for the fully opened and closed state were averaged over five total recording periods of 10 s of each membrane fragment at +30 mV (Figure 8E) and exhibited a significantly longer total open duration for drPanx1b (2.9 ± 0.3 s) compared to drPanx1a (1.4 ± 0.5 s; p < 0.005). drPanx1b constitutes this average by formation of multiple short term events, as indicated by arrows in Figure 8B. Accordingly, drPanx1a channels rest significantly longer (2.5 ± 0.8 s) in the closed state than drPanx1b channels (1.9 ± 0.3 s; p < 0.05). Stepwise depolarization of the holding potential showed that both channels are characterized by a depolarization dependent, exponentially shaped increase in opening probability (Figure 8F). The activation thresholds of the investigated channel proteins differ strongly: drPanx1a was active at holding potentials positive to -45 mV, whereas drPanx1b revealed an activity onset at holding potentials of -60 mV. Evidence for drPanx1 single channel activity close to the resting potential of the cells gives rise for facilitated dye uptake under unchallenged conditions. 

## Discussion

Previously, we described the expression of drPanx1a in horizontal cells and hypothesized a function in ephaptic feedback complementary to Cx52.6 and Cx55.5 [10,12,16]. At that time, no Panx1 labeling was found in the inner retina. Since zebrafish and murine retinae share general structural and functional properties, the apparent lack of Panx1 expression in the inner fish retina was puzzling. The discovery of a second drPanx1 protein, drPanx1b, fills this gap. As suggested by Bond and colleagues (2012), drPanx1 ohnologs most likely originated form a third WGD event implying that pannexin sequences emerged with cartilaginous fish and diverged thereafter. Since in mammals one Panx1 seems sufficient, the question arises how zebrafish benefits from having two drPanx1 proteins with distinct properties, of which drPanx1a is ubiquitously expressed in the brain, like mPanx1, and drPanx1b has a more specialized expression pattern in the CNS [14]. However, in the retina a specialization of both drPanx1 seem to have occurred with drPanx1a expressed in the outer retina and drPanx1b in the inner retina. Having the two *panx1* genes must have been advantageous for teleosts evolution, since the Panx1 could adapt their properties to the specific role they play. For instance, it is interesting to see that drPanx1a is less sensitive to ATP. Recently, evidence has been obtained that retinal horizontal cells release ATP via drPanx1a channels [34]. ATP is hydrolyzed extracellularly leading to acidification of the synaptic cleft. This leads to modulation of the synaptic transmission from cones to horizontal cells. The low ATP sensitivity is fully consistent with this role of drPanx1a. 

### Panx1 proteins are glycosylated with distinct modification patterns

N-glycosylation was reported for murine Panx1 (N254) and drPanx1a (N246) [10,25–27] and is one of the hallmarks of pannexin proteins. Here, we describe additional, putative glycosylation sites in the EL1 (N71, N95) of drPanx1b. Our data suggest that drPanx1b, like mPanx3 (Penuela et al. 2007), is glycosylated at N71. Comparable to mPanx2, which contains an N-glycosylation site at N86 in EL1 but displays only the Gly1 species in western blots [27], we were unable to resolve whether N95 in drPanx1b is glycosylated. However, mutating all three asparagine residues abolished protein expression, emphasizing the importance of amino acid N95 for generation of a functional protein. In general, the entire drPanx1b protein population is extensively glycosylated, a property unique for this protein. Since each asparagine residue within drPanx1b could be modified with different oligosaccharides, complex glycosylation patterns might occur and modulate Panx1 localization and function(s), providing a yet unexplored molecular platform for protein interaction(s) and signaling pathways. It will be a priority in future studies to clarify the relevance of potential interactions with the complex synaptomatrix and whether such interactions modulate neuronal functions in the inner retina.

### Panx1 proteins form Ca^2+^ and pH sensitive channels opening under physiological conditions

Exogenously expressed Panx1 proteins primarily localize in the plasma membrane of N2a cells. Since they lack gap junction-like clustering, it seems that Panx1 form unopposed channels in the plasma membrane. Some Panx1 remained in intracellular compartments. This also happens with connexins and is strongly present in over-expression systems [35]. It was shown that this indicates a population of proteins en route to the cell surface [36]. However, we cannot exclude that Panx1 channels play a role as ER membrane channels [3,37].

All tested Panx1 channels have a basal activity at resting conditions that mediates dye uptake [10,11,38,39]. In agreement with other reports [6,40], elevation of intracellular Ca^2+^ enhances Panx1 activity leading to elevated dye uptake, most probably as consequence of increased Panx1 channel opening or addition of novel channels into the plasma membrane. 

The observed pH dependent channel properties of Panx1 are of particular interest, since physiological pH changes are linked to processes such as development [41], neuronal activity [42,43], lateral inhibition in the outer retina [34,44,45] and the circadian clock [46]. Furthermore, pH-changes are highly relevant for pathological conditions like ischemia and epilepsy *in vivo* [47], and in experimental model systems [48,49]. Therefore, pH dependency of Panx1 channels needs to be carefully revisited and the pH sensor identified. 

### drPanx1 channels are regulated by ATP

ATP acts either excitatory or inhibitory on drPanx1b-mediated dye uptake depending on the concentration used. Application of 3 mM ATP might indirectly activate drPanx1b due to P2X_7_-R activation [5], since the N2a cells used were P2X_7_-R positive. In case of exogenous mPanx1 expression, 500 µM ATP was sufficient to elicit this effect. However, whether Panx1 is truly the pore associated with P2X_7_-R activation is currently under debate [50,51]. The lack of response by drPanx1a channels might be a specific adaptation to their function in the horizontal cell to cone feedback mechanism [34].

The non-canonical ATP binding site of Panx1 is not known yet, but it was reported that ATP binding in mPanx1 involves an arginine residue at position 75 (R75) in EL1 [8,52]. Interestingly, this site is not conserved in the drPanx1a protein used in this study, which enabled us to show that reduction in the number of positively charged amino acids in EL1/EL2 reduced sensitivity to ATP or BBG. Replacement of drPanx1a-C76 with R/K/A/E or drPanx1b-R75 with C/K/A/E partially altered the ATP/BBG sensitivity, confirming ATP sensitivity of Panx1 proteins across species is another conserved property. Differences between this study and the one by Qiu and Dahl (2009) with respect to the observed rank order of ATP/BBG sensitivity of drPanx1a/b mutants can be explained by the different methods used. Since drPanx1 variants with positively charged amino acids at position 75/76 are more sensitive to negatively charged ATP/BBG, we confirm the ATP/BBG mediated inhibition involves the proposed affinity-based interaction with EL1 [8], which was recently shown to be also true for EL2 [52].

### drPanx1 proteins form voltage-gated membrane channels with distinct characteristics

In this study, Panx1 channels were investigated using physiological ion gradients, therewith avoiding harsh ion solutions initially used to characterize Panx1 channels. Further we used a modified preconditioning paradigm recently shown to evoke increased Panx1-mediated currents [33]. For drPanx1b channels, this technical modification evoked large outwards currents at potentials ≥ +30 mV and channel opening at negative potentials. The physiological relevance of this late activation is elusive, but it is tempting to speculate that in a neuronal network an enhanced spiking frequency might potentiate drPanx1b activation. In future studies, it will become necessary to validate whether the large tail current amplitudes and repolarization times in drPanx1b transfectants are due to an extraordinary large or long lasting drPanx1b channel activity or activation of Ca^2+^-dependent currents due to Ca^2+^ influx [53]. 

Similar to mammalian Panx1 (298 - 500 pS), multiple subconductance states and open times were found for drPanx1a and drPanx1b, reflecting complex gating processes [11,32,38]. The conductance decay upon subgate recruitment may reflect narrowing of the channel pore, presumably caused by conformational changes of the transmembrane segments and a spatial redistribution of charges. In addition, it may be possible that partial blocking of the channel pore could involve a dislocation of the C-terminal domain similar to connexin hemichannels and analogous to the ball-and-chain model. Since substates may play a role in the context of the selectivity and permeability filter of Panx1 channels, common coupling of the channel's domains may turn into a more restricted coupling or even give way to selective coupling. Combined quantitative electrical, dye transfer and structural studies will have to elucidate the underlying mechanism(s).

In summary, the cell type specific expression of two Panx1 proteins combined with shared and unique properties suggests that both proteins could operate in distinct neuronal circuits adding functionality to physiological processes shaping visual output. Both channel proteins are located in strategic positions in the inner and outer retina. Shared properties alongside unique properties suggest a potential specialization in function. For instance, drPanx1a seems to be optimized to function in the feedback pathway from horizontal cells to cones in the outer retina where neurons respond with graded potential changes [10,12,34], whereas drPanx1b seems to be optimized for functioning in the inner retina where neurons respond with action potentials. Based on this study, it will become possible to address the roles of the two zebrafish Panx1 proteins in transgenic fish at systems level and in behavioral studies to shed light on the physiological roles of drPanx1a/b *in vivo*. 

## Supporting Information

Figure S1 **Phylogenetic tree of pannexin protein sequences.** Tree was rooted to Hydra innexin (Inx) sequences and bootstrap values are indicated at the nodes. Cx = Connexin; LRRC = leucine-rich repeat–containing, Panx = Pannexin; hm = *Hydra* *magnipapillata*; ce = *Caenorhabditis elegans*; dm = *Drosophila melanogaster*; xt = *Xenopus tropicalis*; gg = Gallus gallus; mm = Mus musculus; hs = Homo sapiens; dr = *Danio rerio*; Cm = *Callorhinchus milii*; on = *Oreochromis niloticus*; ol = *Oryzias latipes*; ga = *Gasterosteus aculeatus*; tr = *Takifugu rubripes*; tn = *Tetraodon nigroviridis*; gm = Gadus morhua; lc = *Latimeria chalumnae*; ac = A*nolis* *carolinensis*; ps = *Pelodiscus sinensis*; mg = *Meleagris gallopavo*; bt = *Bos taurus*; tt = *Taeniopygia guttata*; pt = Pan troglodytes; og = *Otolemur garnettii*; bf = *Branchiostoma floridae*.
(ZIP)

Figure S2 **Sequence alignment of mPanx1, drPanx1a and drPanx1.** The sequence alignment was calculated using CLC Sequence Viewer 6.7 (www.clcbio.com).
(TIF)

Figure S3 **Sequence alignment of drPanx1a and drPanx1.** The sequence alignment was calculated using CLC Sequence Viewer 6.7 (www.clcbio.com).
(TIF)

Figure S4 **Primary characterization of the polyclonal anti-drPanx1a antibody.** N2a cells expressing drPanx1a-EYFP or drPanx1b-EYFP were lysed 48 h post transient transfection. Proteins were detected using the rabbit anti-drPanx1a (A) or mouse anti-GFP (B) antibody and mouse anti-β-actin. The anti-mouse IRDye680 and anti-rabbit IRDye800 antibodies served as secondary antibodies. (**A**) The anti-drPanx1a antibody detects the characteristic three-band pattern (Gly0, Gly1, Gly2, arrows). The Gly0 band has a low intensity. No signal was detected for drPanx1b or for endogenous mPanx1. (**B**) Both drPanx1 proteins can be detected with the anti-GFP antibody, confirming the presence of drPanx1b.
(TIF)

Figure S5 **Correlation between dye uptake and membrane fluorescence in N2a cells expressing EYFP or Panx1 proteins.** N2a cells expressing EYFP or EYFP-tagged mPanx1, drPanx1a or drPanx1b were used for dye uptake assays 48 h post transfection. The plasma membrane fluorescence was correlated with the EtBr fluorescence 5 min after EtBr application. For determining the correlation between membrane fluorescence and dye uptake, the Spearman’s rank coefficient R was calculated using nonparametric correlation (Spearman). Gaussian distribution was not assumed. The confidence limit for significance was 0.05. The result are as followed: R(Spearman) EYFP = 0.2620 (p=0.0021); mPanx1 = 0.7925 (p<0.0001); drPanx1a = 0.7191 (p<0.0001); drPanx1b = 0.8213 (p<0.0001). In all cases a significant correlation was observed, even though the Spearman’s rank coefficient R in EYFP control cells is low. This indicates that the EYFP overexpression may have a slight impact on dye uptake, although the total amount of EtBr uptake does not differ significantly from non-transfected N2a cells. (n = 135).
(TIF)

Figure S6 **Subcellular localization, western blot analyses and dye uptake assays of drPanx1a-C76 and drPanx1b-R75 mutants.** N2a cells expressing drPanx1a-EYFP WT or -C76R, -C76K, -C76A, -C76E mutants or drPanx1b-EYFP WT or -R75C, -R75K, -R75A, -R75E mutants were taken for experiments 48 h post transient transfection. (**A**,**D**) *Subcellular localization of (A) drPanx1a-C76 and (D) drPanx1b-R75 mutants in N2a cells*. The intracellular distribution of (**A**) drPanx1a-C76R, C76K and C76A mutants is similar. All drPanx1a (**A**) and drPanx1b (**D**) mutants showed a clear membrane localization. However, we could also find some cytosolic clustering, with partially huge accumulations, especially in perinuclear areas (except drPanx1a-C76). In contrast, the C76E mutant is mostly found at the plasma membrane with only little proteins found in intracellular compartments. (scale bar = 10 µm) (**B**,**E**) *Western blot analyses of drPanx1a-C76 and drPanx1b-R75 mutants*. The proteins were detected with the anti-GFP, anti-β-actin and the anti-mouse IRDye680 antibodies. (**B**) All drPanx1a mutants display, like drPanx1a WT, the characteristic three-band pattern. C76R and C76K show a more prominent Gly0 signal than the other drPanx1a variants. In addition, the Gly1 bands are more intense than the Gly2 bands, in contrast to the C76E and the WT protein. (**E**) All R75 mutants show, like the drPanx1b WT protein, the characteristic multi band pattern with at least six clearly detectable bands. β-actin served as a loading control. (**C**,**G**) *Dye uptake of drPanx1-C76 of drPanx1b-R75 mutant expressing N2a cells*. EtBr uptake was analyzed 5 min after EtBr (20 µM) application. Each bar represents the mean of EtBr fluorescence + SEM of 135 cells. EtBr uptake of drPanx1 WT expressing cells was set to 100%. (**C**) The reduction of EtBr uptake in C76R, C76K and C76A expressing cells differs significantly from WT levels (p<0.001), in contrast to C76E expressing cells (p>0.05). This effect might be explained either by an impaired channel function caused by the mutations or due to the fact that more C76R, C76K and C76A mutant proteins are retained in cytoplasmic compartments compared to the WT and C76E mutant protein. (**G**) EtBr uptake of investigated mutant drPanx1b-R75 does not differ from drPanx1b WT levels (p>0.05). (n = 135 cells per group; p<0.01 = **; p<0.001 = ***).
(TIF)

Figure S7 **Original traces of membrane current responses before/after preconditioning of EYFP/Panx1 expressing N2a cells.** N2a cells expressing EYFP or EYFP-tagged mPanx1, drPanx1a or drPanx1b were used for whole-cell patch clamp recordings in the voltage clamp mode 48 h post transient transfection. (**A**-**D**) *Original* *traces* *of* *elicited* *membrane* *currents* *of* *transfected* *N2a* *cells* *before* (**I**) *and* after (II) *preconditioning*. The cells were subjected to depolarizing voltage steps ranging from -60 mV to +100 mV before (**I**) and following preconditioning using depolarizing voltage ramps (II) to determine the I/V relation. The arrow in (D) indicates a gain of membrane current response.
(TIF)

Figure S8 **Traces of membrane current responses, I/V relation before/after preconditioning of N2a cells expressing untagged drPanx1b.** N2a cells transfected with pIRES2-mRFP1-drPanx1b, thus expressing tag-less drPanx1b(-), were used for whole-cell patch clamp recordings in the voltage clamp mode 48 h post transient transfection. (**A**) *Original* *traces* *of* *elicited* *membrane* *currents* *of* *N2a* *cells* *expressing* *untagged* *drPanx1b* *before* (**I**) *and* after (II) *preconditioning*. The cells were subjected to depolarizing voltage steps ranging from -60 mV to +100 mV before (**I**) and following preconditioning using depolarizing voltage ramps (II) to determine the I/V relation, respectively. The arrow indicates a gain of membrane current response. (**B**) *I/V relations calculated from the voltage step protocol **I** and **II***. The difference of the I/V relations from **I** and **II** (red line) was calculated by subtraction of the membrane currents of **I** from **II**. Values are mean ± SEM. Preconditioning leads to strongly increased membrane currents evoked at hyper- and depolarized holding potentials. (**C**,**D**) *Comparison* *of* *the* *first* (**C**) *and* *second* (**D**) *voltage* *steps* *between* *tagged and untagged* *drPanx1b* *expressing* *cells*. The difference curves before (C) and after (D) preconditioning were calculated by subtracting membrane currents of N2a cells expressing untagged from ones expressing tagged drPanx1b. In both cases, only slight variations were observed, ruling out that the gain of function after preconditioning is due to the influence of the bulky EYFP tag. (**E**) *Maximum* *current* *amplitudes* *I*_*max*_ *at +100 mV*, (**F**) *maximum* *currents* *amplitudes* *at -60 mV* *and* (**G**) *input* *resistance* *R* *at -60* *mV* *calculated* *from* ***I*** *and* ***II***. Values were calculated from (B). Each bar represents the mean + SEM. For statistical comparison between the values of the different groups obtained as response to the first (depicted in light grey) or second (depicted in dark grey) depolarizing voltage steps, the Kruskal-Wallis test followed by a Dunn’s Multiple comparison post test was performed. For comparison between the values obtained for **I** and **II** within one transfectant group (depicted in black), the Mann-Whitney test was performed. No significant differences were detected by comparing the values obtained for **I** or **II** between both groups for the maximum current amplitudes I_max_ recorded at +100 mV (E). After preconditioning, the maximum current amplitudes are increased highly significantly comparing **I** with **II** in both groups (p<0.0001). Comparing the maximum currents amplitudes recorded at -60 mV for **I** between the groups revealed that untagged drPanx1b(-) expressing cells display significantly higher currents compared to tagged drPanx1b (p<0.01) transfectants (F). Preconditioning prior to **II** lead to highly increased current amplitudes at hyperpolarized membrane potentials in both groups (p<0.001). No significant differences were detected comparing **II** between tagged and untagged drPanx1b. No significant differences were detected by comparing the input resistance R at -60 mV obtained for **I** or **II** between N2a cells expressing EYFP tagged or untagged drPanx1b (G). After preconditioning, the input resistance in **II** decreased highly significantly in both groups (p<0.001). (b = drPanx1b; b(-) = drPanx1b(-); n of I/II: drPanx1b: n = 40/32; drPanx1b(-): n = 29/22; p<0.01 = **; p<0.001 = ***).
(TIF)

Figure S9 **Analyses of membrane currents elicited by depolarizing voltage ramps of EYFP tagged/untagged drPanx1b expressing N2a cells.** N2a cells transfected with pEYFP-drPanx1b or pIRES2-mRFP1-drPanx1b (drPanx1(-)) were used for whole-cell patch clamp recordings in the voltage clamp mode 48 h post transient transfection. Current responses to consecutive depolarizing voltage ramps from -60 mV to +80 mV were recorded within the preconditioning paradigm. (**A**) *Example trace of the current response elicited by 10 s depolarizing voltage ramps in drPanx1b(-) expressing N2a cells*. We found a strong exponential increase in membrane currents at about ≥ +30 to +40 mV holding potentials, reflecting the voltage dependence of the evoked currents. (**B**) *Maximum current amplitudes I_max_ recorded at +80 mV*. (**C**) *Tail* *current* *amplitudes* *I*_*TC*_ *evoked* *after* *rapid* *hyperpolarization* *from +80 mV* *to -60mV* *after* *the* *first* *depolarizing* *voltage* *ramp* *and* (**D**) *time* *at* *which* *the* *tail* *current* *amplitudes* *decreased* *to 50% of its initial value, T*_*1/2*_ *, of* *the* *repolarization* *current*. All values in (B-D) were calculated from the averaged current responses to the first voltage ramp within the preconditioning paradigm. Each bar represents the mean + SEM. No significant differences between drPanx1b and drPanx1b(-) were observed. (drPanx1b: n = 36; drPanx1b(-): 26)).
(TIF)
