# Supplementary material for: Pannexin1 Channel Proteins in the Zebrafish Retina Have Shared and Unique Properties
Source: PLoS One. 2013 Oct 23;8(10):e77722. doi: 10.1371/journal.pone.0077722 (PMC3808535; doi:10.1371/journal.pone.0077722)
Supplement: Figure S1 — Phylogenetic tree of pannexin protein sequences. Tree was rooted to Hydra innexin (Inx) sequences and bootstrap values are indicated at the nodes. Cx = Connexin; LRRC = leucine-rich repeat–containing, Panx = Pannexin; hm = Hydra magnipapillata; ce = Caenorhabditis elegans; dm = Drosophila melanogaster; xt = Xenopus tropicalis; gg = Gallus gallus; mm = Mus musculus; hs = Homo sapiens; dr = Danio rerio; Cm = Callorhinchus milii; on = Oreochromis niloticus; ol = Oryzias latipes; ga = Gasterosteus aculeatus; tr = Takifugu rubripes; tn = Tetraodon nigroviridis; gm = Gadus morhua; lc = Latimeria chalumnae; ac = Anolis carolinensis; ps = Pelodiscus sinensis; mg = Meleagris gallopavo; bt = Bos taurus; tt = Taeniopygia guttata; pt = Pan troglodytes; og = Otolemur garnettii; bf = Branchiostoma floridae. (ZIP) [file pone.0077722.s001.zip › Supporting Material Phylogentic Analsis Sequences.rtf]

Sequences used for phylogenetic calculations:ceInx15			NP_491313ceInx18			NP_741294.1ceInx1			NP_741826.1dmInx6			NP_572374.1dmzpg			NP_648049.1dmInx7			NP_788872.1dmogre			NP_524824.1dmInx3			NP_524730.1hydrInxl3			XP_002155819.1hydrInxl4			XP_002166931.1hydrInxl8			XP_002165350.1hmInxl9			XM_002165314.1hmrInxl10			XP_002155033.1hmInxl1			XP_002154796.1hmInxl6			XP_002170241.1hmInxl5			XP_002170247.1hmInxl11			XP_002160898.1hmInxl7			XP_002166542.1hmInxl2			XP_002160488.1ggLRRC8-3		ggENSGALP00000009943hsLRRC8-3		hsENSP00000350933mmLRRC8-4		mmENSMUSP00000108327mmLRRC8-2		mmENSMUSP00000109284ggLRRC8-2		ggENSGALP00000007267xtLRRC8-2		xtENSXETP00000013413hsLRRC8-1		hsENSP00000338887mmLRRC8-3		hsENSP00000350933mmLRRC8-1		mmENSMUSP00000052055xtLRRC8-1		xtENSXETP00000049449ggLRRC8-1		ggENSGALP00000009947hsLRRC8-2		hsENSP00000359483drCx36.7			NP_001096667hsCx36			NP_065711.1mmCx43			NP_034418xtCx31			NP_001079327.1ggCx32			NP_989702.1lcPanx1			ENSLACT00000005509acPanx1			ENSACAT00000014263xtPanx1			ENSXETT00000014089psPanx1			ENSPSIT00000006060ggPanx1			ENSGALT00000027822mgPanx1			ENSMGAT00000017099ttPanx1			ENSTTRT00000002347btPanx1			ENSBTAT00000015932ogPanx1			ENSOGAT00000015296mmPanx1			NM_019482.2ptPanx1			ENSPTRT00000007801hsPanx1			ENSP00000227638drPanx1a			NP_957210.1onPanx1a			ENSONIT00000009800olPanx1a			ENSORLT00000002661gaPanx1a			ENSGACT00000017521gmPanx1a		ENSGMOP00000002876tnPanx1a			ENSTNIT00000008946trPanx1a			ENSTRUT00000016687gmPanx1b		ENSGMOT00000006144trPanx1b			ENSTRUT00000026807tnPanx1b			ENSTNIT00000009166gaPanx1b			ENSGACT00000025387olPanx1b			ENSORLT00000017033onPanx1b			ENSONIT00000023780lcPanx3			ENSLACT00000010367drPanx3			ENSDART00000027288gmPanx3			ENST00000395842gaPanx3			ENSGACT00000026639trPanx3			ENSTRUT00000006121tnPanx3			ENSTNIT00000011630onPanx3			ENSONIT00000019874olPanx3			ENSORLT00000023812xtPanx3			XP_002940053.1acPanx3			ENSACAT00000000194psPanx3			ENSPSIT00000016310ggPanx3			ENSGALT00000001383mgPanx3			ENSMGAT00000000850btPanx3			ENSBTAT00000012385mmPanx3			NM_172454.2ogPanx3			ENSOGAT00000008030hsPanx3			ENST00000284288ptPanx3			ENSPTRT00000008199ogPanx2			ENSOGAT00000029454btPanx2			ENSBTAT00000037612ptPanx2			ENST00000395842hsPanx2			NP_443071.2mmPanx2			NM_001002005.2ggPanx2			ENSGALT00000000003mgPanx2			ENSMGAT00000008928psPanx2			ENSPSIT00000017077xtPanx2			ENSXETT00000065459acPanx2			ENSACAT00000002295lcPanx2			ENSLACT00000019710trPanx2			ENSTRUT00000011650tnPanx2			ENSTNIT00000011377gaPanx2			ENSGACT00000017828onPanx2			ENSONIT00000013131olPanx2			ENSORLT00000020446drPanx2			ENSDART00000091722gmPanx2			ENSPTRT00000066020drPanx1b		Sequence used for the alignment (not yet in database, see manuscript) MAIARVATEYVFSDFLLKEQSDSKYKGVRLELATDKLVSFIAVGLPLLLISLAFAQEVSVGTQITCFPPTNFTMRQAAYADSFCWAAVEHHPSENETYSAPLHLHKFFPYILLLLAILMYIPALFWRFTAAPSLSSDLSFIMEELDRCYNRAIRLAKSITTKQDKDIAEDPHSGLELTEACFKYPLVEQYLKTKRSSWALAAKYLLCRVLTFLTLLLGCFYLTYYIFWVSPSDQFSCYLRRGILVNQSEVPDVVQCKLVAVGVFRLLSCMNLVVYLLLVPAVVYAGLQPARQHQRGQFLRPYHLLPAFGHVLDLQPATRRYDDLSIYLLFLEENLSELKSYKCLQVLELLSEGGEAAFDTMCLLRTLGQVKTDMVDKRQAQTVNGNPEIVISEIKDVSVLLDDGVQADKSCSCVKDVRQRVVcmPanx2 found on AAVX01203786.1FSLCYTEESIYCYTPNNFTRDQALYARGYCWTELKDAISGVDPSQWPSLFEHKFLPYALLAFGGVMYVPVLGWEFLASTRLTSELNFLLQEIDNCYHRAAEGRAPKIEKQIQSKGPGITEKEKREIIENAEKEKSPEQNLFEKYLERRGQSNFLARLYLGRQLSVVFLSIVPISYLCTYYATQKQNEFTCPLGEPPDMSSGQRQHVSVKCKLPSVQMQRIIVIVDITLLSFINLIILINLVHLFIVRKSNFIFDKLHKVGIKTKKQWQKSQFCDINILALFCNENRDHIKSLNRLDFITNESDLMYDNVVRQLLAALAQSNHDSTPTMHDAGIQTVDPSADPAELDANEQLVIKRPRKKMKWISTTHPLHQPFKDPPTLTKVENHKEKLKPVRRKTVTDSLAAPLLDSGSKCPQDSSSSKNEGYPAANSEKKHSRHFSLDVHPYLLSTKKPKPENQEcmPanx3 (combination of 4 sequences) found on AAVX01216638.1:MSIAGMAAEFILSDSLIREPANTRASSLRLELARDRLIKFISVGLPLLLVSAAFAKEISLGSQISCFPPSNFSTKQAAYVDAICWESLLHQHIQPSGNVAQRSLWIHKSHCWLSVNVDYNLVPADVQVFPYSLLVIAVTMYLPALIWKLFAKPSLASDLIFITDELDKAYNRSIKVAQLIEGVLDFPLLHRYLACKSRSYHLISIYLMRNFLLLVFIAAACLYLIYCHFPAFFQDRFSCSIKSDLLANDPTIPNAIQCKLTSMcmPanx1-1found on AAVX01019061.1:QIFPYVLLLVAILMYIPALFWRFTAAPQLYGDLTFIIEQLDKAYNRGIRLAQHIVSTSGYDcmPanx1-2 found on AAVX01180789.1:FQLLSIINFIVYILLVPVTVYSMLFPLRRNPTFLQVYNLLPQFEVMKMSKGSWNDLTLYLRFLEENVSEIKSHKCLKVLEHVKDMGIAEAMDVLLPLIALGQVKSDVVDBfPanx2-like found on Bf_V2_226  : 72609 - 72713:IDTELPFDKMVKCVTVGVPLFLMSLYFAKEFATASIACFPPQDFGSAHGKYLNVYCWTELKTQPMSLFQHKIFPYILLCIGAIMYLPVVFWNVTAVPTLQAEMSVILTEFDTEKPYAFLLFENYLLCKVKKNQLIRLYLIRQVLVLLGIACTITYLLYFYFENLPDEFSCNIRSGELLVMDGIPDAVNCKLPGVAMFRIACVINIAFY
