# Supplementary figures and images for: Pannexin1 Channel Proteins in the Zebrafish Retina Have Shared and Unique Properties
Source: PLoS One. 2013 Oct 23;8(10):e77722. doi: 10.1371/journal.pone.0077722 (PMC3808535; doi:10.1371/journal.pone.0077722)

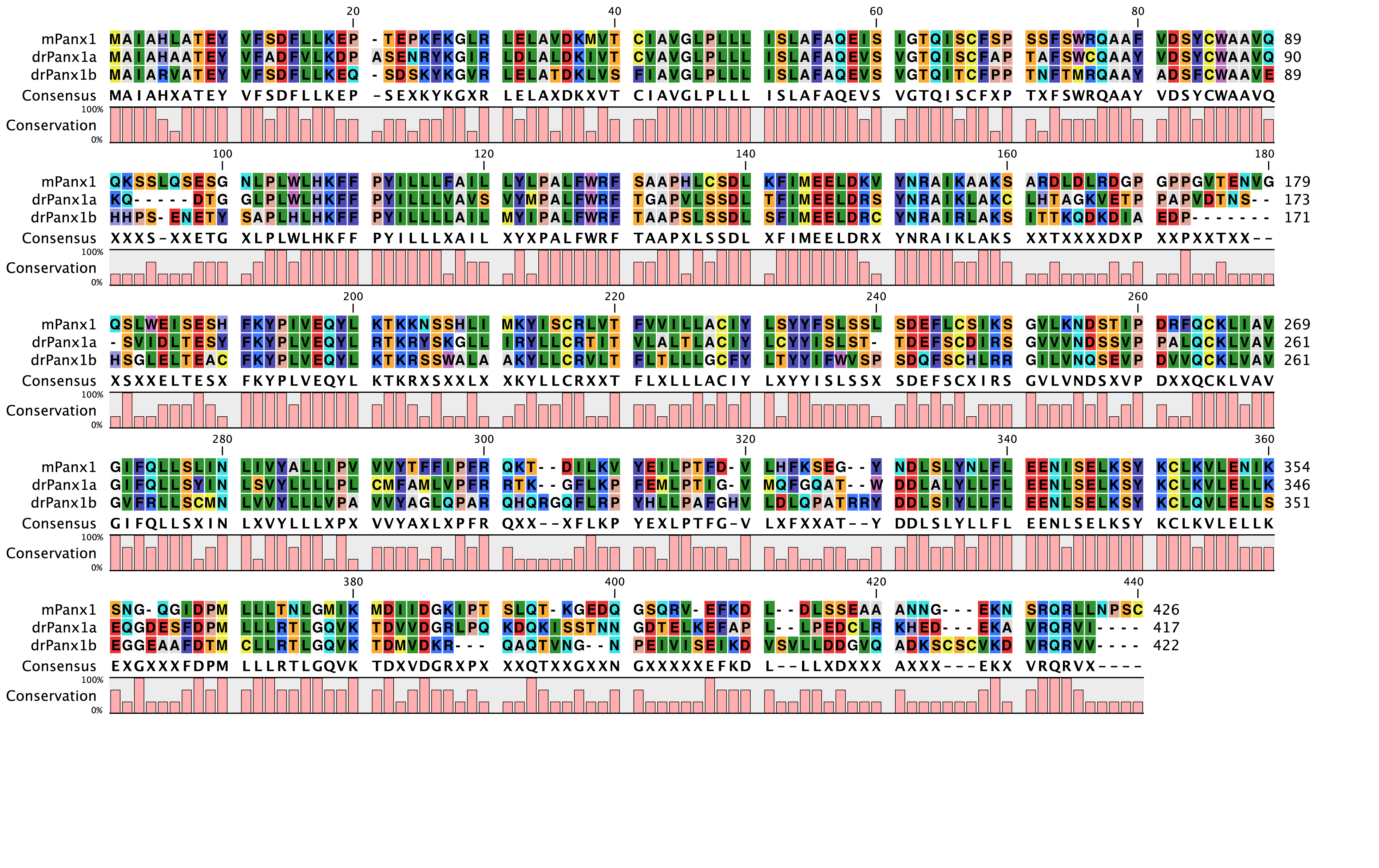

Supplement: Figure S2 — Sequence alignment of mPanx1, drPanx1a and drPanx1. The sequence alignment was calculated using CLC Sequence Viewer 6.7 (www.clcbio.com). (TIF) [file pone.0077722.s002.tif]

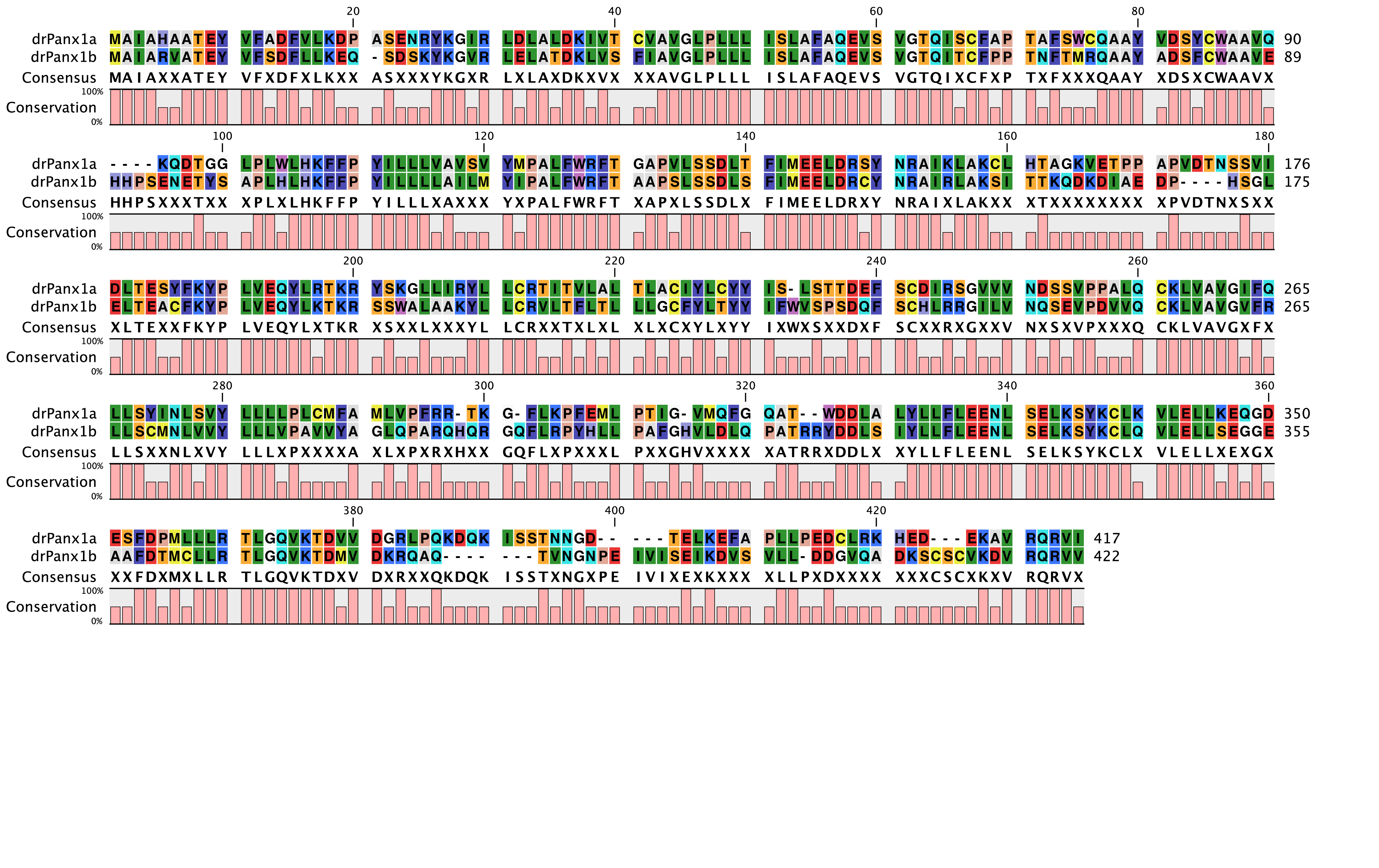

Supplement: Figure S3 — Sequence alignment of drPanx1a and drPanx1. The sequence alignment was calculated using CLC Sequence Viewer 6.7 (www.clcbio.com). (TIF) [file pone.0077722.s003.tif]

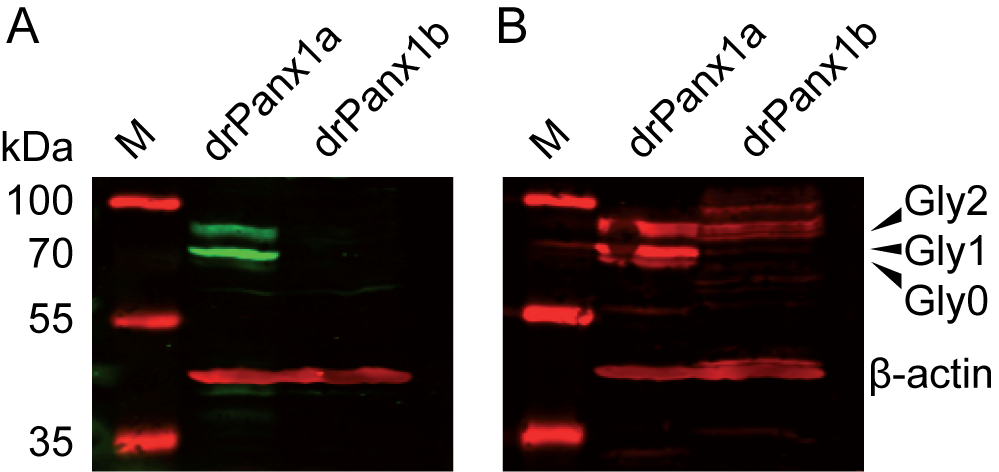

Supplement: Figure S4 — Primary characterization of the polyclonal anti-drPanx1a antibody. N2a cells expressing drPanx1a-EYFP or drPanx1b-EYFP were lysed 48 h post transient transfection. Proteins were detected using the rabbit anti-drPanx1a (A) or mouse anti-GFP (B) antibody and mouse anti-β-actin. The anti-mouse IRDye680 and anti-rabbit IRDye800 antibodies served as secondary antibodies. (A) The anti-drPanx1a antibody detects the characteristic three-band pattern (Gly0, Gly1, Gly2, arrows). The Gly0 band has a low intensity. No signal was detected for drPanx1b or for endogenous mPanx1. (B) Both drPanx1 proteins can be detected with the anti-GFP antibody, confirming the presence of drPanx1b. (TIF) [file pone.0077722.s004.tif]

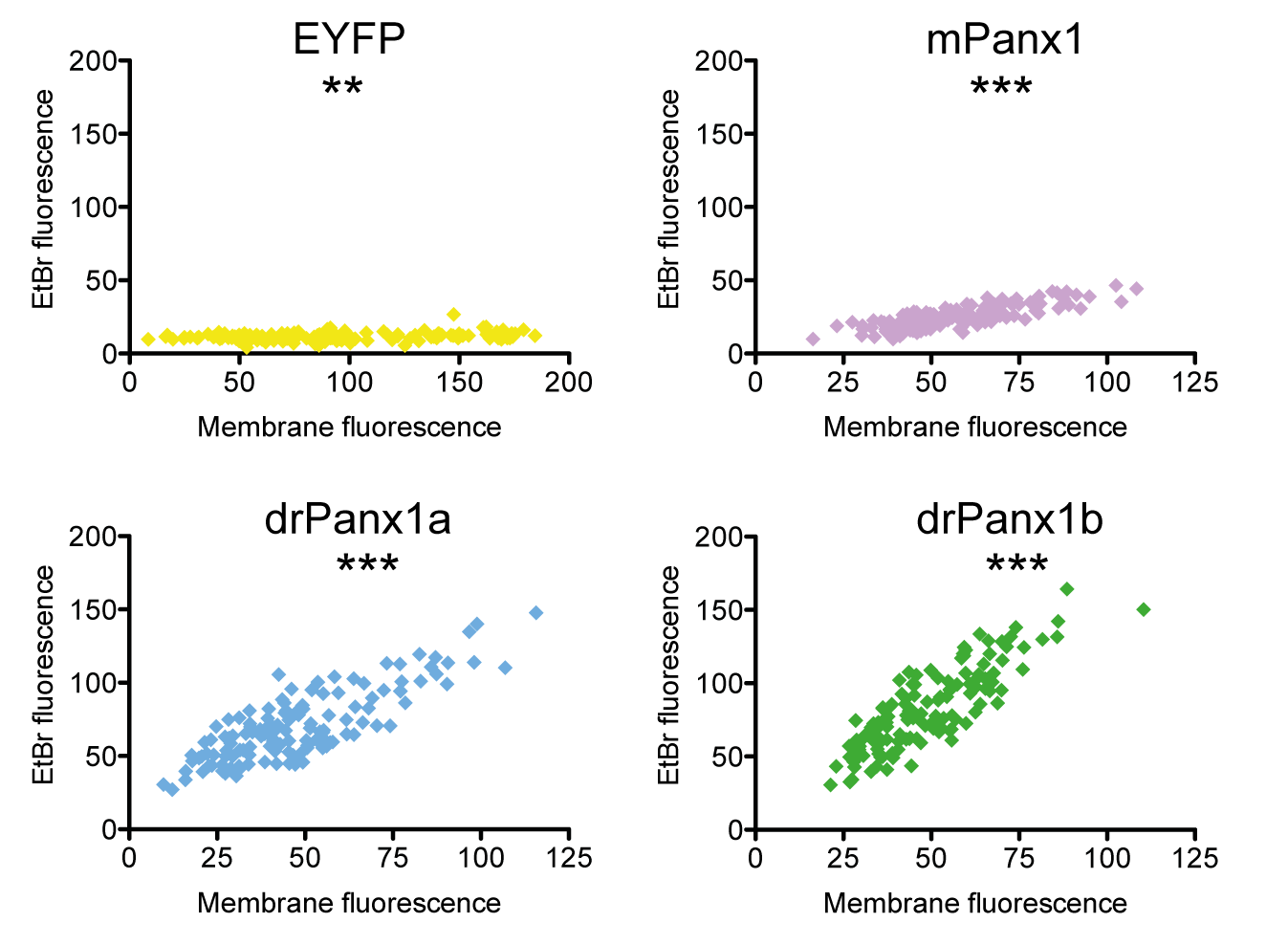

Supplement: Figure S5 — Correlation between dye uptake and membrane fluorescence in N2a cells expressing EYFP or Panx1 proteins. N2a cells expressing EYFP or EYFP-tagged mPanx1, drPanx1a or drPanx1b were used for dye uptake assays 48 h post transfection. The plasma membrane fluorescence was correlated with the EtBr fluorescence 5 min after EtBr application. For determining the correlation between membrane fluorescence and dye uptake, the Spearman’s rank coefficient R was calculated using nonparametric correlation (Spearman). Gaussian distribution was not assumed. The confidence limit for significance was 0.05. The result are as followed: R(Spearman) EYFP = 0.2620 (p=0.0021); mPanx1 = 0.7925 (p<0.0001); drPanx1a = 0.7191 (p<0.0001); drPanx1b = 0.8213 (p<0.0001). In all cases a significant correlation was observed, even though the Spearman’s rank coefficient R in EYFP control cells is low. This indicates that the EYFP overexpression may have a slight impact on dye uptake, although the total amount of EtBr uptake does not differ significantly from non-transfected N2a cells. (n = 135). (TIF) [file pone.0077722.s005.tif]

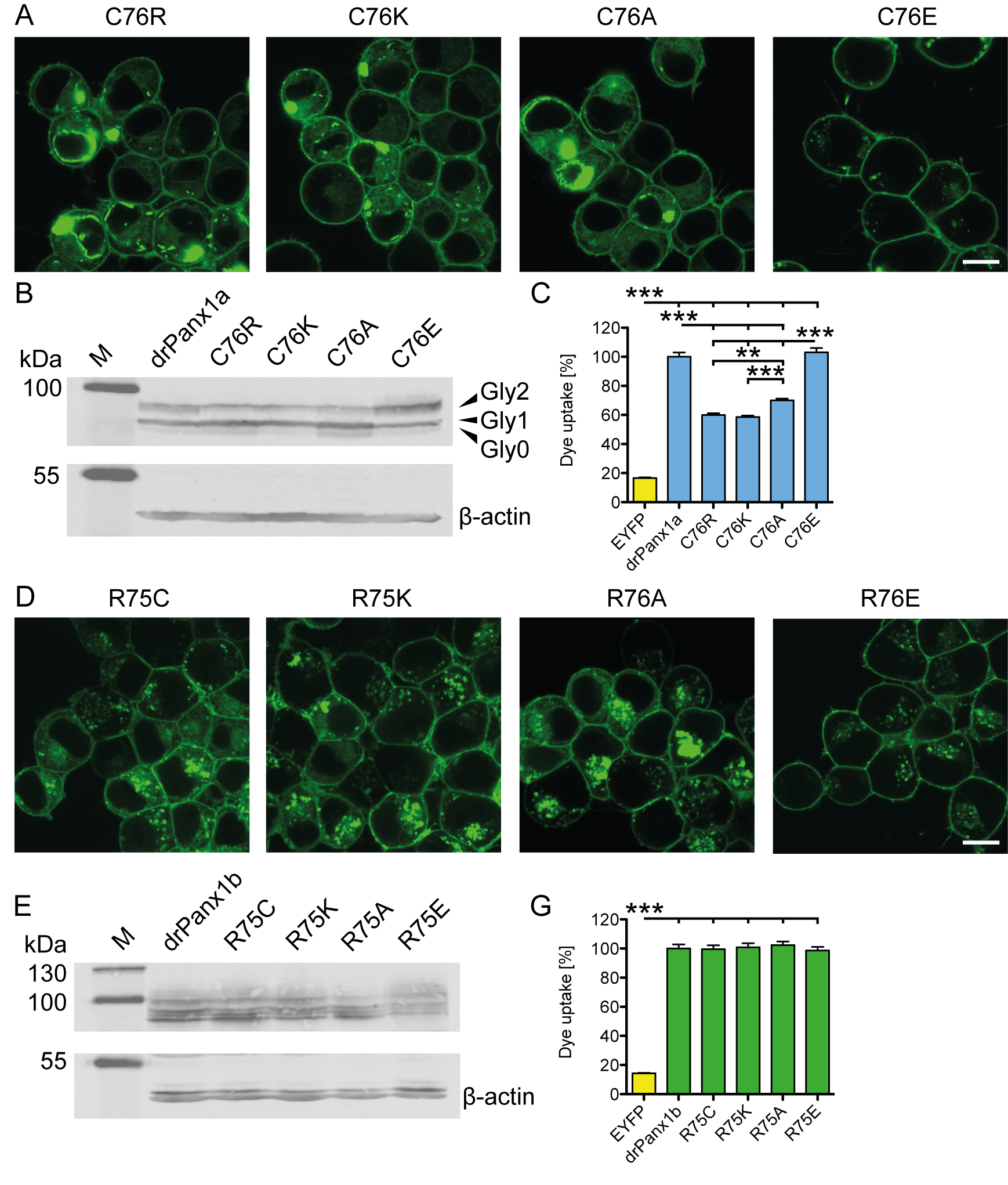

Supplement: Figure S6 — Subcellular localization, western blot analyses and dye uptake assays of drPanx1a-C76 and drPanx1b-R75 mutants. N2a cells expressing drPanx1a-EYFP WT or -C76R, -C76K, -C76A, -C76E mutants or drPanx1b-EYFP WT or -R75C, -R75K, -R75A, -R75E mutants were taken for experiments 48 h post transient transfection. (A,D) Subcellular localization of (A) drPanx1a-C76 and (D) drPanx1b-R75 mutants in N2a cells. The intracellular distribution of (A) drPanx1a-C76R, C76K and C76A mutants is similar. All drPanx1a (A) and drPanx1b (D) mutants showed a clear membrane localization. However, we could also find some cytosolic clustering, with partially huge accumulations, especially in perinuclear areas (except drPanx1a-C76). In contrast, the C76E mutant is mostly found at the plasma membrane with only little proteins found in intracellular compartments. (scale bar = 10 µm) (B,E) Western blot analyses of drPanx1a-C76 and drPanx1b-R75 mutants. The proteins were detected with the anti-GFP, anti-β-actin and the anti-mouse IRDye680 antibodies. (B) All drPanx1a mutants display, like drPanx1a WT, the characteristic three-band pattern. C76R and C76K show a more prominent Gly0 signal than the other drPanx1a variants. In addition, the Gly1 bands are more intense than the Gly2 bands, in contrast to the C76E and the WT protein. (E) All R75 mutants show, like the drPanx1b WT protein, the characteristic multi band pattern with at least six clearly detectable bands. β-actin served as a loading control. (C,G) Dye uptake of drPanx1-C76 of drPanx1b-R75 mutant expressing N2a cells. EtBr uptake was analyzed 5 min after EtBr (20 µM) application. Each bar represents the mean of EtBr fluorescence + SEM of 135 cells. EtBr uptake of drPanx1 WT expressing cells was set to 100%. (C) The reduction of EtBr uptake in C76R, C76K and C76A expressing cells differs significantly from WT levels (p<0.001), in contrast to C76E expressing cells (p>0.05). This effect might be explained either by an impaired channel function c [file pone.0077722.s006.tif]

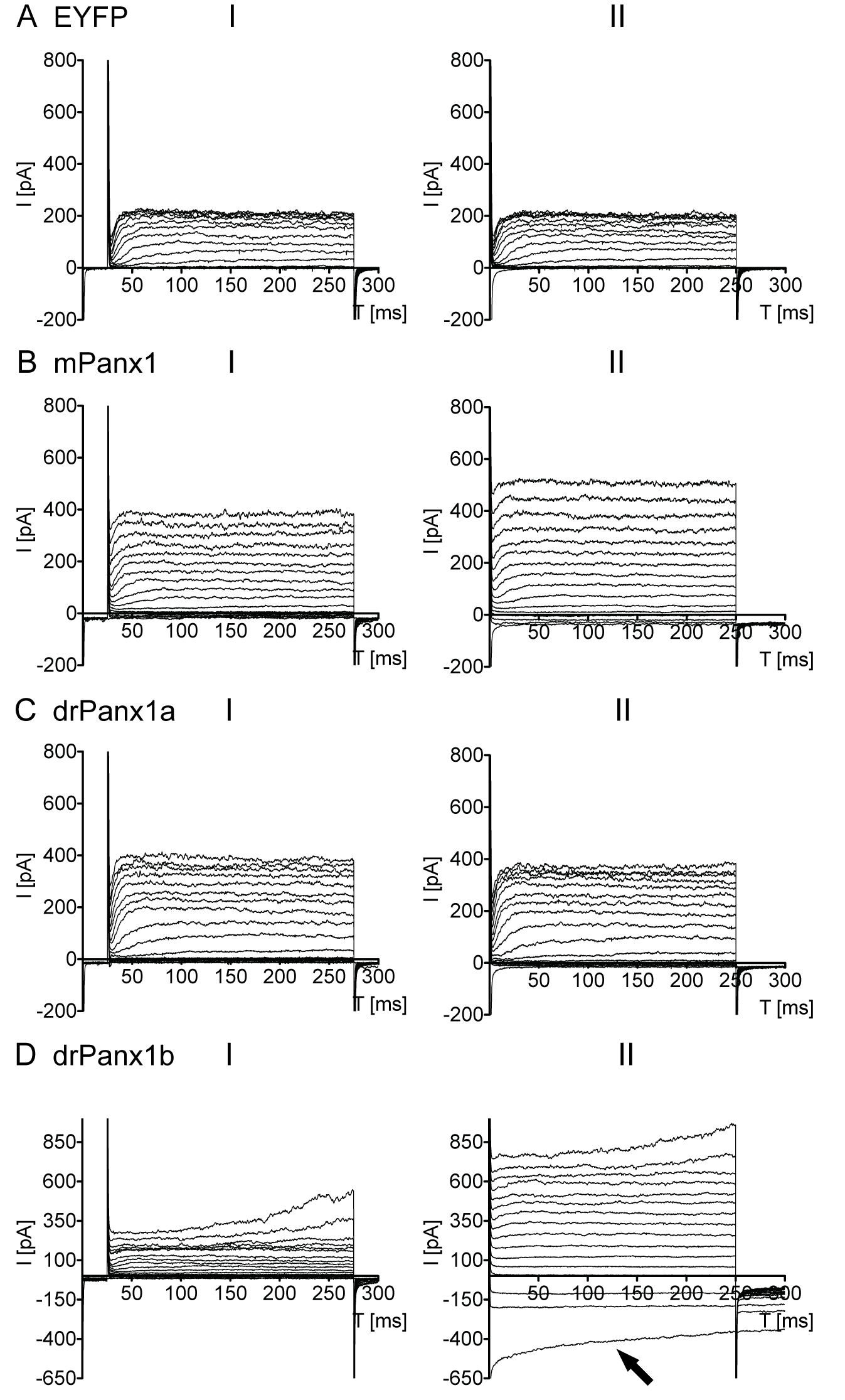

Supplement: Figure S7 — Original traces of membrane current responses before/after preconditioning of EYFP/Panx1 expressing N2a cells. N2a cells expressing EYFP or EYFP-tagged mPanx1, drPanx1a or drPanx1b were used for whole-cell patch clamp recordings in the voltage clamp mode 48 h post transient transfection. (A-D) Original traces of elicited membrane currents of transfected N2a cells before (I) and after (II) preconditioning. The cells were subjected to depolarizing voltage steps ranging from -60 mV to +100 mV before (I) and following preconditioning using depolarizing voltage ramps (II) to determine the I/V relation. The arrow in (D) indicates a gain of membrane current response. (TIF) [file pone.0077722.s007.tif]

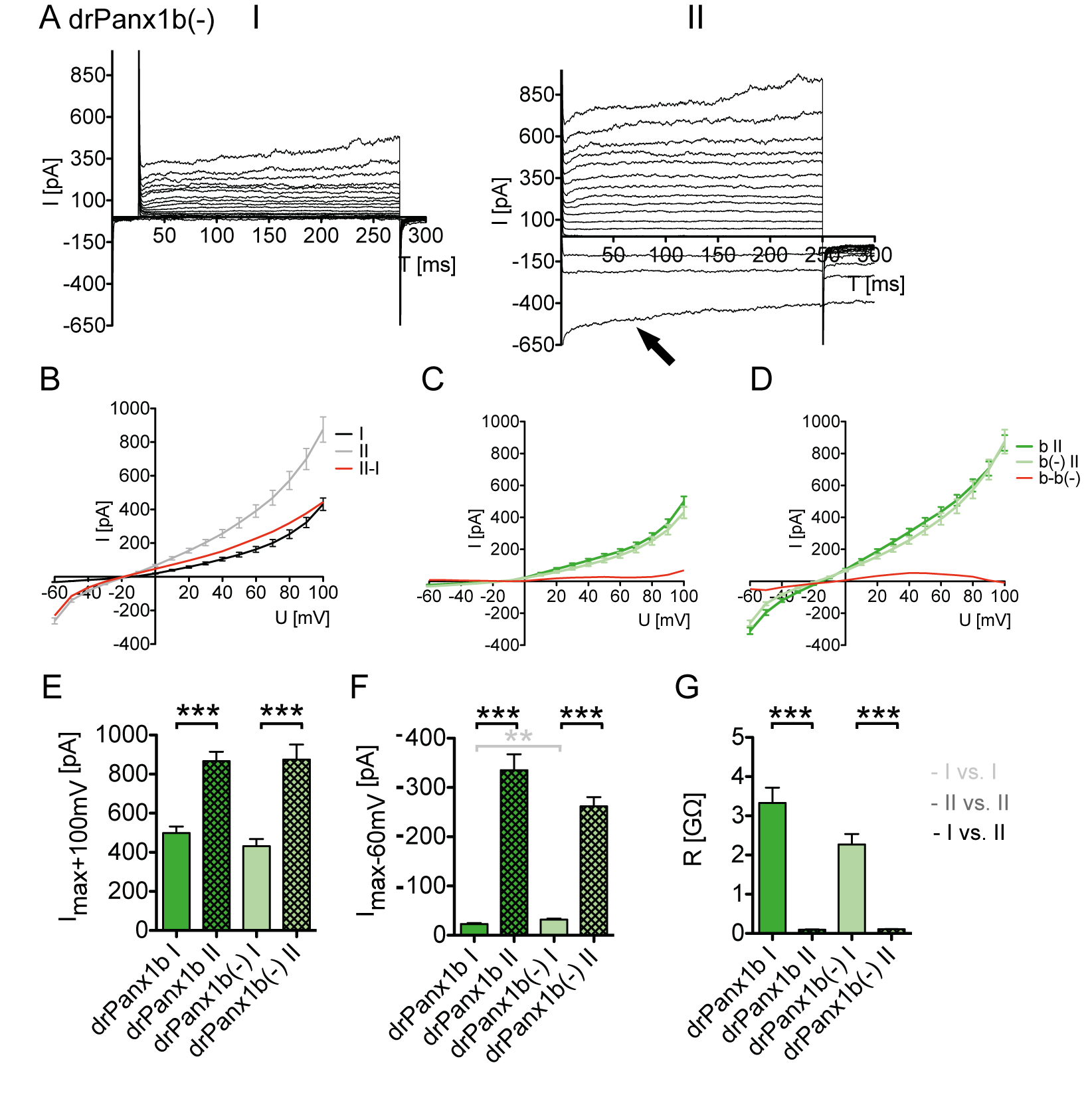

Supplement: Figure S8 — Traces of membrane current responses, I/V relation before/after preconditioning of N2a cells expressing untagged drPanx1b. N2a cells transfected with pIRES2-mRFP1-drPanx1b, thus expressing tag-less drPanx1b(-), were used for whole-cell patch clamp recordings in the voltage clamp mode 48 h post transient transfection. (A) Original traces of elicited membrane currents of N2a cells expressing untagged drPanx1b before (I) and after (II) preconditioning. The cells were subjected to depolarizing voltage steps ranging from -60 mV to +100 mV before (I) and following preconditioning using depolarizing voltage ramps (II) to determine the I/V relation, respectively. The arrow indicates a gain of membrane current response. (B) I/V relations calculated from the voltage step protocol I and II. The difference of the I/V relations from I and II (red line) was calculated by subtraction of the membrane currents of I from II. Values are mean ± SEM. Preconditioning leads to strongly increased membrane currents evoked at hyper- and depolarized holding potentials. (C,D) Comparison of the first (C) and second (D) voltage steps between tagged and untagged drPanx1b expressing cells. The difference curves before (C) and after (D) preconditioning were calculated by subtracting membrane currents of N2a cells expressing untagged from ones expressing tagged drPanx1b. In both cases, only slight variations were observed, ruling out that the gain of function after preconditioning is due to the influence of the bulky EYFP tag. (E) Maximum current amplitudes I max at +100 mV, (F) maximum currents amplitudes at -60 mV and (G) input resistance R at -60 mV calculated from I and II. Values were calculated from (B). Each bar represents the mean + SEM. For statistical comparison between the values of the different groups obtained as response to the first (depicted in light grey) or second (depicted in dark grey) depolarizing voltage steps, the Kruskal-Wallis test followed by a Dunn’s Multiple comparison po [file pone.0077722.s008.tif]

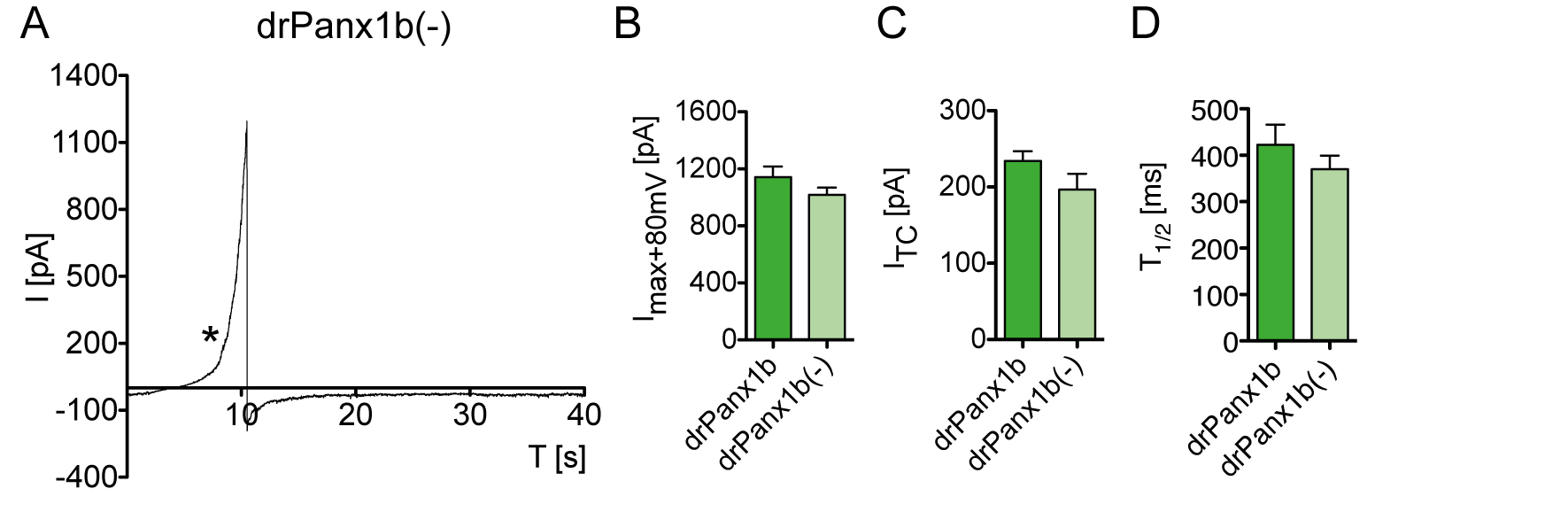

Supplement: Figure S9 — Analyses of membrane currents elicited by depolarizing voltage ramps of EYFP tagged/untagged drPanx1b expressing N2a cells. N2a cells transfected with pEYFP-drPanx1b or pIRES2-mRFP1-drPanx1b (drPanx1(-)) were used for whole-cell patch clamp recordings in the voltage clamp mode 48 h post transient transfection. Current responses to consecutive depolarizing voltage ramps from -60 mV to +80 mV were recorded within the preconditioning paradigm. (A) Example trace of the current response elicited by 10 s depolarizing voltage ramps in drPanx1b(-) expressing N2a cells. We found a strong exponential increase in membrane currents at about ≥ +30 to +40 mV holding potentials, reflecting the voltage dependence of the evoked currents. (B) Maximum current amplitudes Imax recorded at +80 mV. (C) Tail current amplitudes I TC evoked after rapid hyperpolarization from +80 mV to -60mV after the first depolarizing voltage ramp and (D) time at which the tail current amplitudes decreased to 50% of its initial value, T 1/2 , of the repolarization current. All values in (B-D) were calculated from the averaged current responses to the first voltage ramp within the preconditioning paradigm. Each bar represents the mean + SEM. No significant differences between drPanx1b and drPanx1b(-) were observed. (drPanx1b: n = 36; drPanx1b(-): 26)). (TIF) [file pone.0077722.s009.tif]
